# Ancestral origins and invasion pathways in a globally invasive bird correlate with climate and influences from bird trade

**DOI:** 10.1111/mec.13307

**Published:** 2015-08-03

**Authors:** Hazel Jackson, Diederik Strubbe, Simon Tollington, Robert Prys-Jones, Erik Matthysen, Jim J Groombridge

**Affiliations:** * Durrell Institute of Conservation and Ecology, School of Anthropology and Conservation, University of KentMarlowe Building, Canterbury, Kent, CT2 7NR, UK; † NERC Biomolecular Analysis Facility, Department of Animal and Plant Sciences, University of Sheffield, Western BankSheffield, South Yorkshire, S10 2TN, UK; ‡ Evolutionary Ecology Group, Department of Biology, University of AntwerpGroenenborgerlaan 171, Antwerp, 2020, Belgium; § Bird Group, Department of Life Sciences, Natural History MuseumAkeman Street, Tring, Herts, HP23 6AP, UK

**Keywords:** diyabc, invasive alien species, microsatellites, mitochondrial DNA, propagule pressure, ring-necked parakeet

## Abstract

Invasive species present a major threat to global biodiversity. Understanding genetic patterns and evolutionary processes that reinforce successful establishment is paramount for elucidating mechanisms underlying biological invasions. Among birds, the ring-necked parakeet (*Psittacula krameri*) is one of the most successful invasive species, established in over 35 countries. However, little is known about the evolutionary genetic origins of this species and what population genetic signatures tell us about patterns of invasion. We reveal the ancestral origins of populations across the invasive range and explore the potential influence of climate and propagule pressure from the pet trade on observed genetic patterns. Ring-necked parakeet samples representing the ancestral native range (*n* = 96) were collected from museum specimens, and modern samples from the invasive range (*n* = 855) were gathered from across Europe, Mauritius and Seychelles, and sequenced for two mitochondrial DNA markers comprising 868 bp of cytochrome *b* and control region, and genotyped at 10 microsatellite loci. Invasive populations comprise birds that originate predominantly from Pakistan and northern areas of India. Haplotypes associated with more northerly distribution limits in the ancestral native range were more prevalent in invasive populations in Europe, and the predominance of Asian haplotypes in Europe is consistent with the higher number of Asian birds transported by the pet trade outside the native range. Successful establishment of invasive species is likely to be underpinned by a combination of environmental and anthropogenic influences.

## Introduction

Invasive species represent a global concern to society and the wider environment as a consequence of their rapid spread and competitive nature (Holmes & Simons 1996; Chapin *et al*. 2000; Doody *et al*. 2009), transmission of infectious disease (Wikelski *et al*. 2010) and the risks posed by some species to the global agro-economy (Keller *et al*. 2011; Ziska *et al*. 2011; Caplat *et al*. 2012). Invasive species have also been recognized as having detrimental impacts upon native biodiversity, ecosystems and communities (Sakai *et al*. 2001; Allendorf & Lundquist 2003; Gurevitch & Padilla 2004). It is therefore important to understand underlying eco-evolutionary scenarios that allow invasive species to successfully establish and spread outside of their native range so that strategies can be developed to mitigate their future spread.

The ability of a species to establish and become invasive can be influenced by a variety of factors, including propagule pressure (i.e. propagule sizes, propagule numbers, and temporal and spatial patterns of propagule arrival; Simberloff 2009). In general, the higher the propagule pressure, the greater the likelihood of a successful invasion (Cassey *et al*. 2004; Lockwood *et al*. 2005; Blackburn *et al*. 2009). Increased propagule pressure can buffer against environmental and demographic stochasticity and Allee effects that may impede successful establishment, whilst simultaneously mitigating detrimental genetic impacts expected from founding events (Blackburn *et al*. 2015). Alongside this, invasion success is generally higher when species are introduced to climates similar to those occupied in the native range (Williamson 1996; Jeschke *et al*. 2014), and ‘climate matching’ is therefore an important component of assessing risk of invasion by alien species (Thuiller *et al*. 2005). However, reported mismatches between native and invasive ranges, termed climate niche shifts (Guisan *et al*. 2014), necessitate research on the ecological and evolutionary processes that allow species to tolerate climates different from their native ranges (Sexton *et al*. 2002; Kelley *et al*. 2013). To identify how such evolutionary, environmental and human-mediated factors combine to underpin successful invasions, reconstructing pathways of invasion and identifying ancestral source populations are essential prerequisites (Sax *et al*. 2005; Estoup & Guillemaud 2010; Ascunce *et al*. 2011; Lombaert *et al*. 2011; Kirk *et al*. 2013; Perdereau *et al*. 2013).

**Fig. 1:**
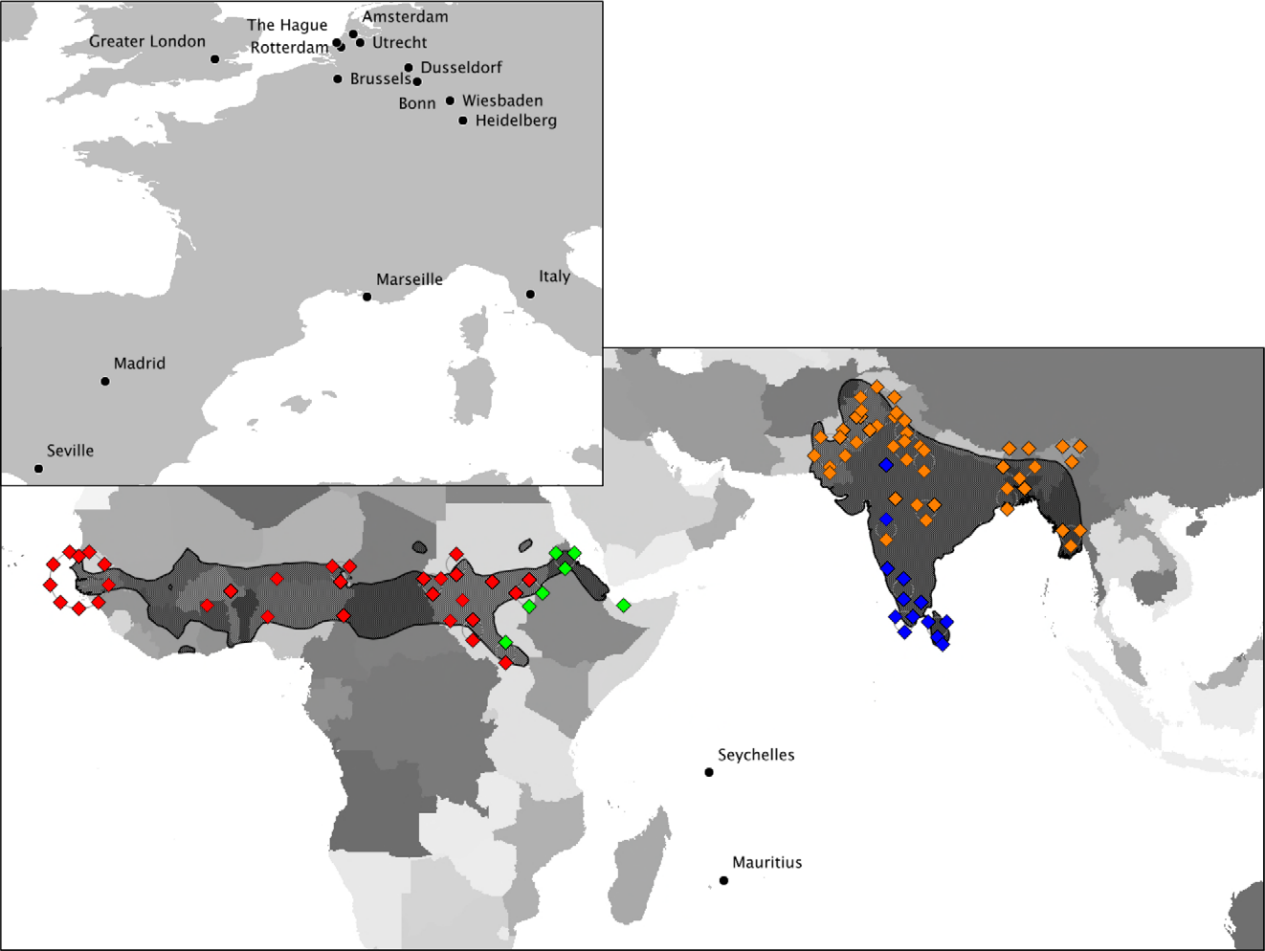
Native range distribution of *Psittacula krameri* [black outlined area across Sub-Saharan Africa and Southern Asia (IUCN 2013)]. Locations of all museum specimens and sampled invasive populations are plotted; where necessary overlapping symbols have been displaced around the true co-ordinate point to display all sample information. Sample locations; diamonds = historical specimens from the native range. Colours refer to the subspecies designation given on the label of each museum specimen; *Psittacula krameri borealis*, orange, *P. k. manillensis*, blue, *P. k. krameri*, red, *P. k. parvirostris*, green; black dots = invasive populations sampled for this study from the invasive range.

In this study, we examine ancestral origins and the influence of invasion pathways (via the pet trade) on patterns of contemporary genetic composition for the globally invasive ring-necked parakeet (*Psittacula krameri*)*,* which comprises multiple invasive populations and a well-documented invasion history. The ring-necked parakeet is one of the most widely introduced parrots in the world, with successful breeding populations established in over 35 countries (Butler 2003; Lever 2005). Native to Asia and sub-Saharan Africa, four subspecies are recognized based on geographical and morphological differences (such as wing, tail, beak and tarsus lengths, Forshaw 2010): two native to Asia (*P. k. borealis*, found in eastern Pakistan, throughout northern India and from Nepal to Burma, and *P. k. manillensis,* from southern India and Sri Lanka) and two in sub-Saharan Africa (*P. k. krameri*, distributed from Senegal to western Uganda and southern Sudan, and *P. k. parvirostris*, from eastern Sudan to northern Ethiopia and Somalia; Fig.1). In their native range, ring-necked parakeets are found in a variety of woodland habitats, farmlands, urban gardens and parks (Juniper & Parr 1998; Khan 2002), whilst in their invasive ranges, they readily colonize forests and parks surrounded by urban habitats (Strubbe & Matthysen 2007). Tests of niche conservatism based on occurrence data and spatial temperature and precipitation gradients (*sensu* Broennimann *et al*. 2012) indicate that across the parakeets' native range, phylogeographic lineages exhibit differing niche requirements (Strubbe *et al*. 2015). These authors also found that the invasion of Europe by ring-necked parakeets is accompanied by a climatic niche shift (*sensu* Broennimann *et al*. 2012; Petitpierre *et al*. 2012) along a temperature gradient. In Europe, parakeets have expanded their climatic niche to colonize environments that are significantly colder than their native range. This scenario meets expectations that endotherms are more likely to tolerate colder conditions in contrast to high temperatures (Sunday *et al*. 2012; Araújo *et al*. 2013).

As one of Europe's top 100 worst alien species (DAISIE, 2008), breeding populations of ring-necked parakeets have become established in a number of European countries since the late 1960s, including the UK, Germany, the Netherlands, France, Spain, Italy, Greece and Belgium (Lever 2005), as well as numerous other countries outside of Europe such as Mauritius and the Seychelles. Such widespread populations of invasive parrots have often been traded extensively due to their popularity as pets (Cassey *et al*. 2004). The rapid spread of this species along with evidence of explosive population growth of parakeets in Europe (Butler *et al*. 2013) presents a cause for agro-economic and environmental concern. These birds are a severe crop pest across their native ranges and are known to decimate maize and fruit crops in India (Ramzan & Toor 1973; Forshaw 2010; Ahmad *et al*. 2012). They have also been shown to compete with native species for nest cavities (Strubbe & Matthysen 2007, 2009a; Hernández-Brito *et al*. 2014) and may have a detrimental impact upon the foraging behaviour of native birds (Peck *et al*. 2014).

This study aims to establish ancestral sources and pathways of invasion for populations of ring-necked parakeets, which have invaded across Europe and the western Indian Ocean. We use mitochondrial (mtDNA) sequence data and a suite of 10 microsatellite DNA markers to examine patterns of genetic structure in the native distribution, and identify the genetic composition of invasive populations across Europe, Mauritius and the Seychelles in relation to their native sources. We then examine the observed genetic patterns to address the following questions: (i) is the genetic composition of the invasive populations a random mix of native genes originating across their Asian and African native range, or (ii) given that during invasion, parakeets have expanded their native climatic niche into colder areas, are genetic patterns associated with temperature differences between native and invasive ranges?, and (iii) can propagule pressure (as measured by records of parakeet imports for the bird pet trade) explain patterns of genetic composition of invasive populations?

## Methods

### Sample collection

Feather and blood samples from ring-necked parakeets were collected from invasive populations in Brussels (*n* = 69), Heidelberg (*n* = 185), Wiesbaden (*n* = 80), Bonn (*n* = 29), Dusseldorf (*n* = 9), Seville (*n* = 57), Madrid (*n* = 2), Greater London (*n* = 195), Tuscany (*n* = 1), Marseille (*n* = 2), Rotterdam (*n* = 75), The Hague (*n* = 13), Amsterdam (*n* = 19), Utrecht (*n* = 2), Mauritius (*n* = 115) and Seychelles (*n* = 2; Table S1, Supporting information). In response to a media campaign, volunteers across Europe collected naturally shed feathers from known roost sites or local parks and gardens. Experienced researchers acquired blood samples from Seychelles and Mauritius. To study ring-necked parakeets from across their ancestral native range, toe-pad samples were collected from 96 museum specimens at the Natural History Museum in Tring, UK (Table S2, Supporting information). Museum specimens were chosen on the basis of their geographic collection location to maximize geographical coverage of samples from across the species' ancestral native range (Fig.1), and contained representatives of each of the four subspecies (subspecies designations were assigned according to each specimens museum label): *Psittacula krameri borealis* (*n* = 45), *P. k. manillensis* (*n* = 13), *P. k. krameri* (*n* = 31) and *P. k. parvirostris* (*n* = 7).

### DNA isolation, amplification and sequencing

Processing of the museum specimens, including DNA extraction and PCR amplification, was carried out in a separate laboratory dedicated to ancient DNA work to prevent contamination. All equipment and surfaces were sterilized before and after each use by irradiation with UV light and application of 10% bleach. Negative controls (where template DNA was replaced with ultrapure ddH_2_O) were included during the DNA extraction and PCR process. A selection of negative extractions PCRs were sequenced to ensure there was no traces of contamination in negative controls. DNA was extracted from both contemporary feather samples and historical toe-pad samples using a Bioline ISOLATE Genomic DNA extraction kit (Bioline, UK). Samples were suspended in 400 μL lysis buffer plus 25 μL proteinase K and incubated at 55 °C overnight (or until the material had completed digested). DNA was washed through a spin column and historical samples were suspended in 50 μL of elution buffer, whilst contemporary samples were suspended in 100 μL of elution buffer. Genomic DNA was extracted from blood using an ammonium acetate precipitation method following Nicholls *et al*. (2000).

Amplification from contemporary DNA samples was conducted for two mtDNA regions: control region using CR19f and CR19r; cytochrome *b* using PKCBf and PKCBr (Table 1). PCR cycling conditions were 94 °C for 1 min followed by 35 cycles of 95 °C for 15 s, 55 °C for 15 s and 72 °C for 10 s and a final elongation step of 72 °C for 10 min. For historical samples, amplification of control region and cytochrome *b* was conducted using a specifically designed suite of overlapping short fragment primers (each between 150–250 bp in length) to provide sequence replication from independent PCRs and detect potential artefacts or contamination (Table 1). Cycle parameters comprised an initial hot start of 95 °C for 1 min followed by 35 cycles of 95 °C/15 s, 52 °C/15 s and 72 °C/10 s followed by a final 10-min 72 °C incubation period. All amplicons were examined by agarose gel electrophoresis to check amplification success and no evidence of contamination in the extraction and PCR negative controls. Amplification volumes of 25 μL contained 2 μL of template DNA, 12.5 μL MyTaq HS Redmix (Bioline, UK), 0.5 μL of each primer and 9.5 μL of ddH_2_0. PCR product was purified and amplified using a 3730 ×L DNA analyser (Macrogen Inc.). Sequences were edited in four Peaks (Griekspoor & Groothuis 2005) and aligned in Clustal (Larkin *et al*. 2007). Manual edits were made in Jalview (Waterhouse *et al*. 2009). The two genes were concatenated using Sequence Matrix (Vaidya *et al*. 2011).

**Table 1:** Suite of mtDNA PCR primers used to amplify cytochrome *b* and control region fragments in historical and contemporary *Psittacula krameri* specimens

Primer name	Sequence (5′–3′)
Historical specimen primers	
Cb1f	CTA CCA TTC ATA ATC ACC AGC C
Cb1r	GTG AGG GAG AGG AGT ATG ATA G
Cb2f	CTA TCA TAC TCC TCT CCC TCA C
Cb2r	TAG GAT CAG TAC GGA GGC AG
Cb3f	AAC AAC TCC CCC ACA CAT C
Cb3r	CGG CGA GTG TTC AGA ATA G
CR1f	CGT TCG TGT TTG CTT ACA TTT C
CR1r	GGT CCG TGT TGT TTG TTT TG
CR2f	CAC TGA TGC ACT TTT TCT GAC
CR2r	GGT GAA ATG TAA GCA AAC ACG
MCR2f	GAT GCA CTT TTT CTG ACA TCT G
MCR2r	GTT TCT TGA AAT GAA TCA CAG
CR3f	GAA CAA ACA AAC GTC TCC TTC
CR3r	GGA TAT TTG AGT GCG AGT GAC
Contemporary specimen primers	
PKCBf	CGGCCTACTCCTAGCCGCCC
PKCBr	GGGAAGCAGGCCGGAAGGC
CR19f	CACAGGCTCATTTGGTTCGC
CR19r	TAAGCTACAGGGACATTCGGGG

We used a suite of 21 microsatellite markers available for *Psittacula* parakeets that have been shown to cross-amplify in ring-necked parakeets (Raisin *et al*. 2009). PCR protocols followed Raisin *et al*. (2009), optimized at a total volume of 2 μL. Each PCR contained 1 μL (blood) or 3 μL (feathers) ≈ 10 ng/μL of DNA that was air-dried, 1 μL of primer mix (fluorescently labelled forward) at 0.2 μm and 1 μL QIAGEN Multiplex PCR Master Mix (QIAGEN Inc). PCRs were conducted using differently fluorolabelled forward primers (HEX and 6-FAM; Raisin *et al*. 2009). PCR cycling conditions were 95 °C for 10 min followed by 35 cycles of 93 °C/30 s, 52 °C/90 s and 72 °C/90 s, with a final incubation at 72 °C for 10 min. PCR was performed with a low annealing temperature (52 °C) to increase the likelihood of amplification (Primmer *et al*. 2005). Individuals were sex-typed using the Z-002B marker (Dawson 2007) to check for loci that were sex-linked. PCR products were separated using an Applied Biosystems 3730 DNA Analyser with ROX™ 500 as a size standard. Alleles were identified and scored using genemapper 3.7 (Applied Biosystems, UK). Three loci were ambiguous to score and therefore were excluded from the analysis (*Peq06*, *Peq08* and *Peq09)*. Loci identified as sex-linked in echo parakeets (*Psittacula echo*) by Raisin *et al*. (2009) were confirmed to be sex-linked in ring-necked parakeets based on a complete lack of heterozygotes in females and therefore also excluded (*Peq16* and *Peq21*), resulting in a total of 16 loci. Due to the degraded nature and low volume of the DNA extracted from historical museum specimens, only 10 of the 16 loci amplified (those with the largest allele size failed to amplify). Each sample was genotyped twice to ensure consistent scoring of alleles (genotypes which could not be scored consistently were removed), and to identify potential genotype errors. Such degraded samples are known to be susceptible to genotyping errors due to allelic dropout (Taberlet *et al*. 1996; Hoffman & Amos 2005; Wandeler *et al*. 2007). For comparative analysis between museum and contemporary microsatellite genotype data sets, the contemporary data set was condensed to the same 10 loci that were genotyped in the museum specimens. Deviations from Hardy–Weinberg equilibrium and null allele frequencies at each locus were estimated using cervus. Evidence of genotyping errors (allelic dropout and stuttering) was assessed using microchecker 2.2.3 (Van Oosterhout *et al*. 2004).

### Characterization of population origin using Bayesian methods

First, a median joining haplotype network (Bandelt *et al*. 1999) was constructed in popart (Leigh *et al*. 2015; software available at: http://www.popart.otago.ac.nz), to infer relationships between haplotypes from the ancestral native and invasive ranges. Haplotype frequencies and distributions in the ancestral native and invasive range were then plotted on a map using qgis (QGIS Development Team 2014).

A Bayesian clustering approach was utilized using the program structure v2.3.4 (Pritchard *et al*. 2000) to determine the most likely number of clusters (*K*) across the ancestral native range using the microsatellite data from the 10 selected loci. Ten repeated runs were conducted with *K* ranging from one to 10 for 500 000 iterations with a burn-in of 100 000, under the admixture model and specifying correlated allele frequencies (Falush *et al*. 2003), with prior information included to assist clustering (The analysis was conducted twice including either subspecies or location as prior information; Hubisz *et al*. 2009). The assignment values, log likelihood scores and Δ*K* were evaluated using structure harvester (Earl & von Holt 2011) to infer the most likely number of genetic clusters. clumpp (Jakobssen & Rosenberg 2007) was used to obtain proportion averages across the multiple runs, and these assignment probabilities were mapped using qgis. To infer proportions of genetic ancestry from each ancestral native source, a further Bayesian clustering approach was utilized to assign individuals in invasive populations to reference ancestral native source populations, using the program structure v2.3.4 (Pritchard *et al*. 2000). Once the most likely number of clusters in the ancestral native range was identified (*K* = 2; Asia and Africa), a further admixture analysis was performed assuming the same two putative ancestral native source populations for the assignment of invasive populations. Here, the ancestral native clusters were set as ‘known’ by activating the ‘*usepopinfo’* flag, and the invasive populations were included as unknown. This approach requires the analysis to cluster the invasive populations with their ancestral native source populations (Pritchard *et al*. 2000) and was conducted separately for each invasive population. clumpp (Jakobssen & Rosenberg 2007) was used to obtain proportion averages across the multiple runs, and these assignment probabilities were plotted using distruct (Rosenberg 2004) and mapped using qgis.

**Fig. 2:**
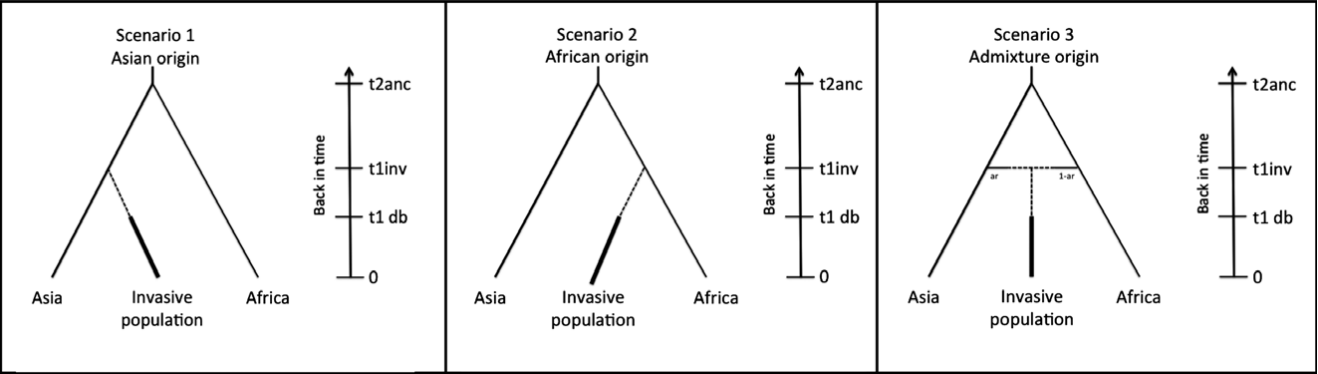
Graphical representation of the three scenarios of ancestral origin examined using approximate Bayesian computation implemented in diyabc. Scenario 1 corresponds to a direct ancestral origin from Asia, scenario 2 corresponds to a direct ancestral origin from Africa, and scenario 3 corresponds to an admixed origin from both Asia and Africa. In each case the black arrow indicates backwards steps in time in generations to when invasive populations were founded (t1inv generations ago), and then back in time to when the native populations diverged (t2anc). Duration of bottlenecks from founding events are included at t1 db. In scenario 3, the admixture rate (ar) represents the genetic contribution of the native populations.

We used approximate Bayesian computation methods, executed in diyabc v2.04 (Cornuet *et al*. 2014), to infer the most likely ancestral source for each of the invasive populations of ring-necked parakeets. Populations with very small sample sizes (*n* < 2) were excluded (Madrid, Marseille, Utrecht, Seychelles and Tuscany), leaving 11 populations in the final diyabc analysis (Brussels, Heidelberg, Wiesbaden, Bonn, Dusseldorf, Seville, Greater London, Rotterdam, The Hague, Amsterdam and Mauritius). Three competing scenarios were designed where samples from the ancestral native range data were clustered into two main ancestral sources according to our structure results (*K* = 2) and geographic location, Asia and Africa. Our scenarios tested whether invasive populations of ring-necked parakeet originate solely from Asia (scenario 1), solely from Africa (scenario 2) or are an admixture, originating from both the Asian and African ancestral native range (scenario 3) (Fig.2). Each scenario was characterized by a number of historical and demographic parameters that were expressed as the number of generations back in time. We assumed a generation time of 5.6 years based on a genetically confirmed social pedigree from more than 20 years of individual-based life history data of the ring-necked parakeets' closest extant relative, the Mauritius parakeet, *Psittacula echo* (Tollington *et al*. 2013). Invasive populations were founded ‘t_1_inv’ generations ago [based on the first recorded breeding populations of ring-necked parakeets across Europe in the late 1960s (Butler *et al*. 2013) and on Mauritius in the 1880s (Cheke & Hume 2008)], with a bottleneck duration of ‘db’ generations, before achieving a larger established effective population size within 1–10 generations, and had an effective number of founders ‘N_1_b’ between 1 and 100. Priors for bottleneck duration and the effective number of founders were broad as no prior information is available. The ancestral native African population diverged from the Asian population ‘t_2_anc’ generations ago (based on molecular dating by H. Jackson, D. Strubbe, R. Pryce-Jones, E. Matthysen, & J. J. Groombridge, in prep). Scenario three includes an admixture event where the admixture rates ‘ar’ and ‘1-ar’ represent the genetic contribution of each ancestral native population (see Table 2 for details).

**Table 2:** Prior distributions of historical and demographic parameters for scenarios modelled using diyabc

	Parameter name	Distribution	Min–Max values
Invasive population	N1	Uniform	1–10 000
Ancestral Asia	N2	Uniform	10–10 000
Ancestral Africa	N3	Uniform	10–10 000
Time of invasion	t1inv	Uniform	1–15 (*Mauritius = 1–40)
Duration of bottleneck	db	Uniform	1–10 (*Mauritius = 1–30)
Effective number of founders	N1b	Uniform	1–100
Ancestral divergence	t2anc	Uniform	200 000–300 000
Rate of admixture	ar	Uniform	0.001–0.999

Each parameter is expressed as the number of generations back in time assuming a generation time of 5.6 years. Invasive populations were founded ‘t1inv’ generations ago, with a bottleneck duration of ‘db’ generations and an effective number of founds ‘N1b’. The native African population diverged from the Asian population ‘t2anc’ generations ago. Scenario 3 includes an admixture event where ‘ar’ represents the genetic contribution of each native population.

Analyses for each invasive population were performed using a combined data set of both mtDNA sequences and microsatellite genotypes. A total of 3 × 10^6^ simulated data sets were used with uniform prior distributions of *N*_e_ and bottleneck durations (Table 2). A stepwise-mutation model was assumed for microsatellites with default parameters (Estoup *et al*. 2002), and a HKY model of evolution was applied for mitochondrial markers as identified in PartitionFinder (Lanfear *et al*. 2012). We used summary statistics for each population and each population pair including the mean number of alleles, mean expected heterozygosity (Nei 1987) and mean size variance for both one and two sample summary statistics for microsatellites. For mtDNA sequences, we used Tajima's D (Tajima 1989) as a one sample summary statistic and *F*_ST_ (Hudson *et al*. 1992) as a two sample summary statistic. Pre-evaluation of each scenario was performed by PCA (Principal Component Analysis) within diyabc, and to identify the best scenario, we used diyabc's logistic regression to compare posterior probabilities across 500 simulated pseudo-observed data sets. Performance of the scenario parameters was assessed by using diyabc's functions for estimating type I (false positives) and type II (false negative) errors, and by computing the relative bias across the 500 pseudo-observed simulated data sets. Finally, to verify the goodness of fit for each scenario, model checking was implemented in diyabc to compare summary statistics between observed and simulated data sets.

### Drivers of contemporary genetic composition within invasive populations

To place in context the resulting genetic data and patterns of invasion by ring-necked parakeets alongside the potential contribution to these patterns by releases or escapes of pet parakeets, all import records of ring-necked parakeets between 1975 and 2007 from the ancestral native ranges (Asia and Africa) were obtained for Spain, Italy, UK, Germany, Netherlands, Belgium and France from the cites Trade Database (CITES 2014). No equivalent trade information was available for the genetically sampled populations on Mauritius or Seychelles. The limited available cites data only allowed for a simplistic measure of propagule pressure based on number of imports per European country from Asia and Africa. Chi-square tests were used to verify whether the distribution of Asian vs. African mtDNA haplotypes detected across Europe differed from that expected by propagule pressure, whereby distributions in the invasive range ought to reflect relative quantities of parakeet imports from Asia and Africa (cites trade data). To compare the observed genetic composition of European parakeet populations derived from microsatellite data with expectations based on propagule pressure, the genetic composition of all populations within a country was averaged, as cites trade data are only available at the country level. Using the proportion of Asian/African birds documented as having been imported into each European country, and the proportion of Asian/African genes observed from the genetic data for each country, a binomial GLM was applied to test for differences between the proportions of imported and observed Asian/African genes.

### Genetic patterns and temperature differences between ancestral native and invasive ranges

During the invasion of Europe, ring-necked parakeets have shifted their climatic niche towards colder areas (Strubbe *et al*. 2015). As sample sizes are too limited to carry out haplotype-level tests of niche conservatism (*sensu* Broennimann *et al*. 2012), we apply two alternative tests to verify whether the distribution of haplotypes between and within invasive European parakeet populations is associated with temperature differences between ancestral native and invasive ranges. Given the species-level niche shift along a temperature gradient, haplotypes characterized by a lower cold niche limit in the ancestral native range can be expected to be more successful at invading colder parts of Europe. Using the maximum latitude at which haplotypes occur in the ancestral native range as a proxy for tolerance of cold temperatures, and focusing on the predominant Asian haplotypes, the following tests were performed. First, the maximum native-range latitude of haplotypes was correlated with their prevalence across European populations (Table S3, Supporting information). Second, a weighted average maximum native-range latitude for each European population was calculated (i.e. weighing the maximum native-range latitude of each haplotype present in a European population according to its prevalence in that population). This weighted average latitude was then correlated with the latitude of the European population, to test the hypothesis that haplotypes associated with colder temperatures in the ancestral native range will be more prevalent in northern European parakeet populations.

## Results

### Ancestral origins of invasive populations

A total of 868 bp of DNA sequences were obtained from the mtDNA control region spanning the hypervariable domain I and conserved domain II (522 bp), and cytochrome *b* (346 bp) gene for 696 specimens from the invasive ranges (Table S1, Supporting information) and 96 museum native range specimens (58 from Asia and 38 from Africa). Owing to the fragile nature of the DNA derived from museum samples, partial sequences (196–868 bp) were obtained for some specimens. A total of 819 specimens from the invasive range were genotyped at 10 microsatellite loci (Table S1, Supporting information), and 92 specimens from the native range (56 from Asia and 36 from Africa). No linkage disequilibrium was detected between pairs of microsatellite loci following Bonferroni corrections (Rice 1989). Deviations from Hardy–Weinberg equilibrium were detected at two of the 10 loci across all populations combined (*Peq*02 and *Peq*15). Estimated frequencies of null alleles ranged between 0.003- and 0.084% per locus. The museum specimen genotyping error rates were 3.89%, and the estimated frequencies of null alleles per locus ranged between 0.003 and 0.460%.

**Fig. 3:**
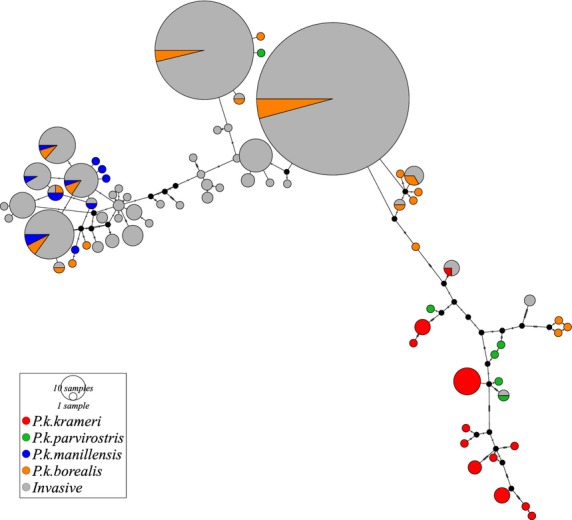
Median joining haplotype network comprising 44 mitochondrial (mtDNA) haplotypes shared between the native and invasive ranges, and an additional 30 haplotypes observed from the invasive range. Haplotypes from the invasive distributions are in grey. Native haplotypes are coloured according to the subspecies designation given on the label of each museum specimen; Asian subspecies = *Psittacula krameri borealis,* orange, and *P. k. manillensis*, blue; African subspecies = *P. k. krameri*, red, and *P. k. parvirostris,* green. Circles are proportional to haplotype frequency.

**Fig. 4:**
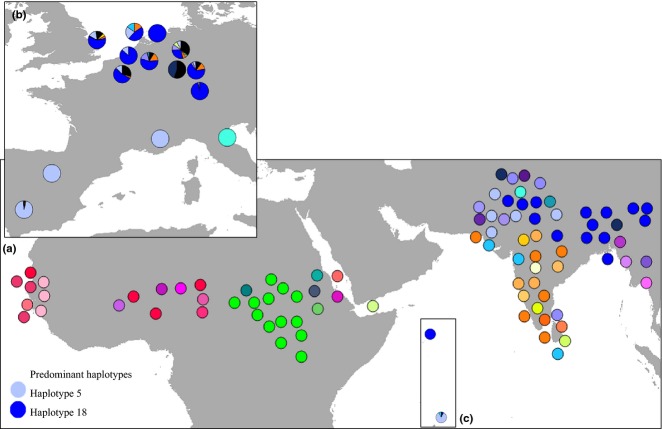
Distribution of 74 haplotypes across the native and invasive range of *Psittacula krameri,* based on 868 bp of mitochondrial DNA (mtDNA). (a) pie charts represent the approximate location of each museum specimen. Different colours relate to different haplotypes. (b and c) pie charts represent the frequency of native mtDNA haplotypes detected within each invasive population across Europe, Mauritius and Seychelles (for observed frequencies for each population, see Table S4, Supporting information). Black proportions indicate mtDNA haplotypes detected in invasive populations that were not detected in the native range. Locations of pie charts represent the approximate geographical location of the samples (exact coordinates/location details are given Table S2, Supporting information).

In total, 44 unique haplotypes were identified from the 96 museum samples representing ring-necked parakeets across their ancestral native range. When the mtDNA sequence data derived from the ancestral native and invasive populations were combined, a total of 74 haplotypes were identified (Table S4, Supporting information). A total of 14 haplotypes were shared between both the ancestral native and invasive ranges. However, 30 haplotypes were found only in the invasive populations and were not detected in the ancestral native range. These haplotypes mainly clustered with haplotypes found in the Asian ancestral native range (Fig.3). The majority of haplotypes shared between the ancestral native and invasive range were of Asian origin, predominately from Pakistan and northern regions of India (Fig.4). Of the 14 invasive haplotypes identified from the ancestral native range, 12 were of Asian origin (comprising 602 sequenced parakeet individuals), whereas only two were of African origin (comprising four sequenced parakeet individuals); a haplotype from Ethiopia was detected in an individual from the Greater London population, and a haplotype from Sudan (Darfur) was detected in two Greater London individuals and in an individual from the Heidelberg population in Germany. In contrast, a single common haplotype detected in numerous locations in Asia was frequently detected across the invasive range. This haplotype was found in high frequencies in Bonn (37.9%), Brussels (58.4%), Seychelles (100%), Heidelberg (95.8%), Wiesbaden (67.5%), Amsterdam (84.2%), Utrecht (100%), Rotterdam (51.3%), The Hague (46.1%) and Greater London (59.9%). Invasive populations in Seville, Madrid and on Mauritius all predominately shared a second haplotype from Asia (Asia_Punjab_5), which was also detected in the Greater London population (14.9%).

A structure analysis using ancestral native range microsatellite data only revealed a Δ*K* statistic suggesting two distinct population clusters, one in Asia and one in Africa (*K* = 2; Evanno *et al*. 2005). Assignment probabilities indicated that all individuals from the ancestral range (apart from a single African specimen which was assigned to the Asian cluster) were unambiguously assigned to one of the two clusters (cluster 1; *q* = 0.816–0.982, cluster 2; *q* = 0.857–0.990), with each cluster corresponding to these two continental ranges (*K* = 2, using subspecies prior; ln*K* = 12.65 and Δ*K* = 8.36, using location prior; ln*K* = 50.54 and Δ*K* = 9.62). These results were used to infer genetic clustering for use in our diyabc modelling. Using approximate Bayesian modelling, implemented with diyabc, scenario 3 was the best-supported model under a logistic regression (posterior probability >0.9) in all populations (Table 3), indicating these invasive populations most likely derive from an admixture of both ancestral Asian and African sources. The type I error from scenario three across all invasive populations was 0%, whilst combining all simulations across the two alternative scenarios resulted in a type II error varying between 0 and 2.8% (Table 3). In support of scenario 3, the observed summary statistics were generally concordant with the simulated data set (Table S5, Supporting information), and the priors were not biased (Table S6, Supporting information).

**Table 3:** Posterior probabilities (confidence intervals) and type I and type II errors in support of scenario 3, modelled in diyabc, for each invasive population of ring-necked parakeet

Invasive population	Posterior probability (CI)	Type I error	Type II error
Brussels	1.0000 (1.0000, 1.0000)	0.00	0.002
Heidelberg	0.9999 (0.9998, 1.0000)	0.00	0.01
Wiesbaden	0.9997 (0.9991, 1.0000)	0.00	0.01
Bonn	0.9998 (0.9989, 1.0000)	0.00	0.008
Dusseldorf	0.9934 (0.9824, 1.0000)	0.00	0.028
Seville	0.9998 (0.9992, 1.0000)	0.00	0.006
Greater London	0.9998 (0.9989, 1.0000)	0.00	0.004
Rotterdam	1.0000 (1.0000, 1.0000	0.00	0.01
The Hague	0.9447 (0.8273, 1.0000)	0.00	0.006
Amsterdam	0.9997 (0.9989, 1.0000)	0.00	0.014
Mauritius	0.9948 (0.8945, 1.0000)	0.00	0.014

**Fig. 5:**
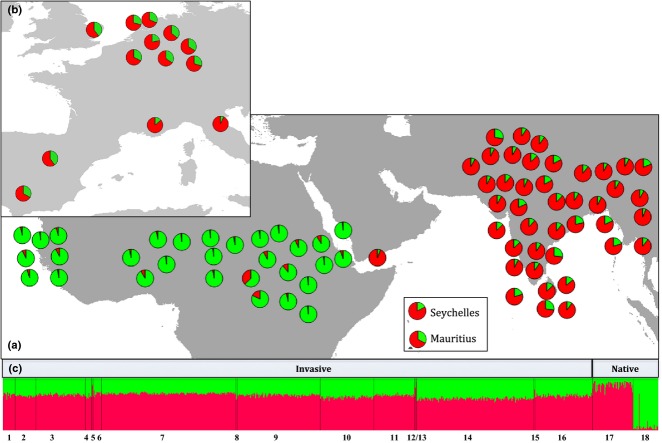
(a) Genetic clustering of ring-necked parakeet from their native range using a Bayesian clustering approach in structure and 10 microsatellite loci, to determine the optimal number of native range clusters (*K* = 2). (b) Assignment of invasive ring-necked parakeets to their putative native ancestral sources using ‘*popflaginfo*’ option in structure. (c) structure output, groups labelled 1–16 are from invasive ranges, groups labelled 17 and 18 are from the native range. From left to right; 1. Amsterdam, 2. Bonn, 3. Brussels, 4. Dusseldorf, 5. Marseille, 6. The Hague, 7. Heidelberg, 8. Madrid, 9. Mauritius, 10. Rotterdam, 11. Seville, 12. Seychelles, 13. Tuscany, 14. Greater London, 15. Utrecht, 16. Wiesbaden, 17. Asia, 18. Africa.

Our additional analysis of the microsatellite data for each invasive population using structure [i.e. the analysis whereby we assumed two putative ancestral source populations (Asia, Africa)] also supports the approximate Bayesian computation scenario, and these results were used to infer the proportions of ancestry from each ancestral native source population for each of the invasive populations. All invasive populations demonstrated a higher assignment probability to the Asian cluster, suggesting a stronger Asian (rather than African) contribution to the genetic make-up of the invasive populations (Brussels = 68%, Heidelberg = 71%, Wiesbaden = 66%, Bonn = 66%, Dusseldorf = 64%, Seville = 67%, Madrid = 60%, Greater London = 60%, Tuscany = 94%, Marseille = 86%, Rotterdam = 63%, The Hague = 71%, Amsterdam = 69%, Utrecht = 78%, Mauritius = 68% and Seychelles = 83%; Fig.5).

### Genetic patterns and information from the bird pet trade

Between 1984 (the earliest ring-necked parakeet trade record available from cites) and 2007, before the EU ban on the trade of wild birds was implemented (Commission Regulation (EC) No.318/2007), a total of 109 467 ring-necked parakeets were imported from the Asian ancestral native range and 37 072 from the African ancestral native range (Table 4) into the EU countries included in this study (the proportions equate to 74.7% from Asia and 25.3% from Africa). Imports from Africa into Europe were only received from Senegal, whereas imports from Asia were received from a wider geographic source including India, Pakistan, Sri Lanka and Bangladesh. The source composition of imported parakeets also varied considerably between countries; for example, all imports into France were from Senegal (1053 parakeets), whilst all Asian imports to Spain and Italy were from Pakistan (48 036 and 45 316 parakeets, respectively). Greater London, Germany and France received larger numbers of ring-necked parakeets from Senegal than from Asia.

**Table 4:** Total number of ring-neck parakeets imported into countries in the invasive range between 1984–2007 (CITES 2014)

Importer	Total global imports	India	Pakistan	Sri Lanka	Bangladesh	Total imports from Asian range	Total imports from African range (Senegal)
UK	16 520	4607	18	0	2	4627	10 396
Netherlands	7206	4277	1500	4	0	5781	201
Germany	11 967	2479	753	0	0	3232	7682
Belgium	5639	2469	2	0	0	2471	2020
France	1620	0	0	0	0	0	1053
Italy	53 167	0	45 316	0	4	45 320	3556
Spain	62 334	0	48 036	0	0	48 036	12 164

Imports from the African range are all from Senegal. No import data is available for Mauritius or Seychelles.

A comparison of the observed distribution of haplotypes detected across Europe (12 Asian and 2 African haplotypes) against an equal expectation (seven Asian, seven African) suggests that Asian haplotypes are more common across Europe than expected (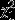 = 7.14, *P* < 0.01). However, accounting for the known skew in propagule pressure (from Asia: 109 467 birds imported (75%) vs. from Africa: 37 072 birds (25%), Table 4) indicates (after rounding) that the corrected expected proportion is 10 Asian vs. four African haplotypes, and against this null expectation, the observed difference fails to reach significance (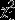 = 1.40, *P* = 0.24). No relationships were observed between microsatellite-based estimates of the proportion of Asian/African genes observed in European countries and the proportion of Asian/African birds imported into each country (GLM coefficient = 0.121, SE = 0.764, *t* = 0.159, *P* = 0.880).

Compared to expectations based on propagule pressure, Asian genes made up a larger than expected percentage of the population in Italy (propagule pressure: 93% Asian imports vs. 94% Asian genes observed in the population), the UK (31% imports vs. 60% genes), Germany (30% imports vs. 67% genes), Belgium (55% imports vs. 68% genes and France (0% imports vs. 86% genes). The proportion of Asian genes detected was lower than expected in Spain (80% imports vs. 64% genes) and the Netherlands (97% imports vs. 70% genes).

### Genetic patterns and temperature differences between ancestral native and invasive ranges

**Fig. 6:**
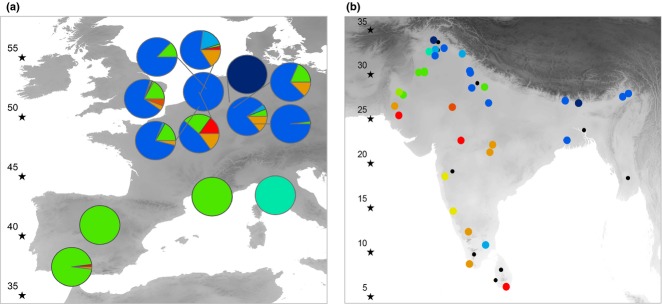
(a) Occurrence and frequency of native Asian mitochondrial (mtDNA) haplotypes in Europe according to their (b) native Asian range maximum cold niche limits. Specimens belonging to mtDNA haplotypes with high maximum latitudes (i.e. cold-tolerance) are depicted in blue. Specimens belonging to haplotypes present in Asia but not detected in Europe are shown by smaller black dots. The shaded background of each map indicates minimum temperature of the coldest month (darker shading represents colder temperatures). Black stars indicate latitude gradient.

Haplotypes with a higher maximum native-range latitude (i.e. associated with colder temperatures in the ancestral native range) are more prevalent in invaded Europe (*r*_23_ = 0.58, *P* < 0.01; haplotype prevalence across Europe log transformed). This result holds when comparing the maximum native-range latitude of haplotypes detected in Europe with haplotypes not detected (*t*_17.11_ = 3.74, *P* < 0.01). The weighted average maximum native-range latitude of haplotypes present in a European parakeet population was positively and significantly correlated with the latitude of that population (*r*_12_ = 0.54, *P* < 0.05; Fig.6). Yet, despite our efforts to collect as much data as possible from southern European populations, our data set is biased towards more northern populations (i.e. eight northern European populations vs. four southern). Moreover, of the four southern populations, all but Seville have limited sample sizes. We therefore carried out a jackknife resampling in which each southern European parakeet population was omitted from the data set. Our findings that haplotypes with higher maximum native-range latitude are more prevalent in invaded Europe and that haplotypes detected in Europe have higher maximum native-range latitudes than haplotypes not detected are robust to jackknife resampling. However, the correlation between the weighted average maximum native-range latitude of haplotypes present in a European parakeet population and the latitude of that population is only robust to the removal of the Tuscany population. The correlation fails to achieve statistical significance when omitting any of the other southern European parakeet populations (Table S7 and S8, Supporting information). Lastly, it should be noted that in our data set, latitude is strongly correlated with temperature (correlation between minimum temperature of the coldest month (derived from Hijmans *et al*. 2005) and latitude: *r* = 0.94, *P* < 0.0001). Replacing latitude with temperature consequently does not affect our results (Table S9, Supporting information).

## Discussion

By combining extensive sampling across native and invasive geographical ranges, this study is the first to establish the genetic ancestral origins of a global avian invader, the ring-necked parakeet. Both mtDNA and nuclear markers reveal the Asian native range to be the predominant ancestral source for the invasive populations established across Europe and the Indian Ocean islands. This observed genetic pattern in the invasive range is consistent with propagule pressure, in the form of parakeets imported from the ancestral native Asian vs. African ranges for the pet trade. Our results also indicate that, in Europe, haplotypes originating from northern Asia occur in higher frequencies, which may be explained by smaller differences in temperature between the ancestral native and invasive ranges of these haplotypes. Interestingly, 30 haplotypes were identified in the invasive ranges but not in the ancestral native ranges. We suspect that these haplotypes may be present in the native ranges but were not detected by our sampling (96 native samples vs 696 invasive samples); alternatively, they may be a result of admixture between ring-necked parakeets originating from different ancestral native continents. Below, we discuss the supporting evidence for each alternative explanation and their likely levels of influence on the observed patterns.

### Genetic patterns and temperature differences between ancestral native and invasive ranges

Strubbe *et al*. (2015) found that intraspecific climatic niche variation is present among native-range ring-necked parakeet phylogeographic lineages and that including this niche variation into ecological niche models (which are statistical techniques that link the occurrence of species to aspects of the environment; Elith *et al*. 2006) improves the accuracy of predictions of parakeet occurrence across Europe. Here, we show that the most prevalent mtDNA haplotypes in the invasive populations either have a wide ancestral native distribution, spanning almost the entire Indian subcontinent or originate from across Pakistan and the northern areas of India, the native range of *P. k. borealis*. As parakeets carrying these haplotypes tolerate colder parts of the ancestral native range, our results suggest that their higher prevalence across Europe [and the higher accuracy of ecological niche model forecasts accounting for intraspecific niche variation found by Strubbe *et al*. (2015)] can potentially be explained by a higher establishment success and subsequent survival of individuals with these haplotypes in the cooler parts of the parakeets' invasive range. Theory predicts that because of physiological limits, for endotherms, tolerance to cold is more likely than tolerance to high temperatures (Sunday *et al*. 2012; Araújo *et al*. 2013). Indeed, given the temperature differences between ancestral native and invasive ranges, parakeets have not been introduced to areas warmer than their ancestral native range. Even parakeets with haplotypes from the coldest parts of the ancestral native range experience, in their native range, temperatures similar to those of the warmer parts of the invasive range (such as Seville, Mauritius and Seychelles). The fact that haplotypes associated with warmer parts of the ancestral native range (i.e. southern Asia) are more frequently found in more southern and thus warmer invasive populations (Seville) suggests that for these haplotypes, temperature differences with the more northerly invasive populations in Greater London, Netherlands, Belgium and Germany may be too large to tolerate.

Several eco-evolutionary scenarios have been proposed to explain the invasiveness of populations. Evolutionary changes can occur independently at each introduction location, adaptation may take place at an initial site of invasion with other areas subsequently invaded from this site or alternatively, and key evolutionary changes for invasion may arise in the ancestral native range, before introduction in the invaded range (Hufbauer *et al*. 2012). For example, by combining ecological niche modelling techniques with common-garden experiments and genetic data, Rey *et al*. (2012) showed that the invasion of Mediterranean Israel by the tropical ant *Wasmannia auropunctata* could be explained by adaptation to cold at the southern limit of the ancestral native range before introduction to Israel. Such an invasion scenario, termed ‘prior adaption’ by Hufbauer *et al*. (2012; not to be confused with pre-adaption, which implies a change of function), may also explain the invasion of ring-necked parakeets, as our results point to a prior adaptation of certain haplotypes to colder conditions in the northern parts of the parakeets' ancestral native Asian range.

Yet, given our limited sampling of southern European parakeet populations, more research is needed to conclusively rule out alternative explanations. More data on the distribution of parakeet haplotypes across Europe are needed to run meaningful ecological niche models (*sensu* Hernandez *et al*. 2006) aimed at delineating candidate geographical source areas for haplotypes across the ancestral native range. Such models could provide another independent line of evidence for climatic similarities between ancestral native and invaded regions as driver of parakeet invasion success in Europe. European parakeet populations experience climates colder than their ancestral native range, but experimental common-garden experiments for verifying whether cold adaptation occurred during invasion are impractical with such a long-lived vertebrate species. However, recent advances in genomic methods permit using genomic signatures of selection to identify genes associated with adaptation during invasion and in response to differencing climates, without requiring common-garden experiments (Chown *et al*. 2014). Future population sampling for genetic analyses should focus specifically on parakeet populations established within geographical regions identified by ecological niche models. Genome-wide analyses of both contemporary and historic (i.e. from museum specimens) ring-necked parakeet DNA allow assessing the temporal dynamics of evolutionary change during invasion (Smith *et al*. 2011), as well as elucidating demographic footprints of past climate change in native populations (Miller *et al*. 2012). Such analyses can strongly improve the inference of invasion history and provide a long-term perspective for understanding how selection pressures during invasion impact species invasion success (Hoffmann & Sgrò 2011; Chown *et al*. 2014).

### Evidence for an influence of bird trade

Analyses of nuclear and mtDNA evidences a predominantly Asian origin for the invasive populations of ring-necked parakeets, which is consistent with expectations from the bird pet trade, as about 75% of all ring-necked parakeets imported in Europe originate from Asia. Cassey *et al*. (2004) reported that successful colonizations of invasive parrots are most likely to be attributed to those species that are traded and kept as pets. Recent research suggests that wild-caught parrots demonstrate acute stress responses, behaviours which can make them more adept at escaping from captivity and surviving in novel wild environments, thus contributing to their success as an invasive species (Carrete & Tella 2008; Cabezas *et al*. 2012). Strubbe & Matthysen (2009b) found that most ring-necked parakeet populations in Europe stem from unintentional escapes of cage birds, supporting the use of the proportion of Asian vs. African birds imported as (an admittedly rough) proxy of propagule pressure. Similarly, Russello *et al*. (2008) and Edelaar *et al*. (2015) found that geographic origins of monk parakeets (*Myiopsitta monachus*) introduced across the US were concordant with trapping records from the native range, suggesting that propagule pressure exerted by the international pet bird trade contributed to the distribution of genetic diversity across the monk parakeets' invasive range.

At the European level, genetic patterns are thus in line with expectations based on propagule pressure, but interestingly, some countries, such as the UK, received much larger numbers of imports from Africa compared to other countries, yet this difference is not reflected in their mtDNA and nuclear composition. Conversely, Seville, the warmest European parakeet population, exhibits a larger amount of African genes than expected based on trade patterns. Such mismatches may be due to the fact that populations such as Greater London were established prior to the earliest available trade records (ring-necked parakeets were recorded as breeding in 1971; Balmer *et al*. 2013), to stochastic genetic processes such as founder effects or genetic drift, or may alternatively be caused by Asian and African ring-necked parakeets differing in their prior adaptation to one or more aspects of the environment (e.g. climate, association with human disturbance, breeding phenology).

Whilst our findings demonstrate that the total number of parakeets imported into European countries influences the current genetic makeup of European parakeet populations, recent studies employing transport network topology analyses (i.e. which account for factors such as yearly fluctuations in number of species imported) have shown that trade can also influence biological invasions in more subtle manners (e.g. see Banks *et al*. 2015). To facilitate such further analyses, we provide more detailed cites data used in this study in Tables S10 and S11 (Supporting information). Lastly, it should be noted that to fully unravel how eco-evolutionary scenarios lead to the emergence of invasive populations, higher resolution data on the origin of source populations and propagule pressure may be needed. For example, although we have no evidence to assume this, theoretically speaking, it could be possible that the higher prevalence of north Asian haplotypes in Europe is not due to a prior adaptation to colder environments, but to larger amounts of northern vs. southern Indian parakeets introduced and escaped in Europe. cites trade data are only available at the country level, and this crude proxy for propagule pressure thus does not allow testing for such a scenario.

## Conclusions

Our study has provided the first substantial insights into the ancestral origins of ring-necked parakeets across their widespread invasive range. Our findings provide strong evidence that the invasive populations predominately comprise birds of Asian ancestry, in particular from the northern areas of the ring-necked parakeets' range in Asia. Our data also suggest that a combination of nonrandom effects from prior adaptation to cold in the northern parts of the ancestral native Asian range, along with high levels of trade in birds, has influenced the signatures of ancestral origin within the invasive populations of ring-necked parakeets. This proposed eco-evolutionary scenario, shaped by a large number of importations from Asia and subsequently influenced by prior adaptation to cold climates in the ancestral native range, represents an important first step in reconstructing pathways of invasion. Our identification of the ancestral origins of invasive ring-necked parakeets provides crucial information that can be applied to further studies such as ecological niche models aimed at predicting areas at risk of invasion by this species, and ultimately to inform decision-makers tasked with developing future policy of invasive alien species. Based on our findings, such policy should, in addition to controlling global trade of wild birds, also incorporate measures to control or prohibit the commercial breeding and keeping of parrots as pets in regions outside of their native ranges. Our findings provide an important contribution for understanding the evolutionary adaptation of a globally invasive species in a novel environment.

## Data accessibility

Mitochondrial and microsatellite data: Dryad entry doi:10.5061/dryad.c8t42.

## Supporting Information

**Table S1** Sample size and location information for invasive populations included in this study. **Table S2** Sample and location information for historical specimens from the Natural History Museum in Tring, UK. **Table S3** Latitudes for native range Asian haplotypes and their frequency in invasive European populations. **Table S4** Haplotype frequencies for each of the 74 mtDNA haplotypes discovered across the native and invasive ranges. **Table S5** Model checking for scenario 3 in diyabc based upon 10^6^ simulated data sets. **Table S6** Measures of performance; mean bias and RRMISE in support of scenario three in diyabc based upon 10^6^ simulated data sets. **Table S7** Concerning the analysis of proportion of haplotypes, the low sample size of southern populations makes our results dependent on the Seville population. **Table S8** Sensitivity' tests on our analyses of haplotypes prevalence or presence–absence in invaded Europe. **Table S9** About the use of latitude as a proxy for temperature.

**Table S10** cites data; imports from Asia per year. **Table S11** cites data; imports from Africa per year.
