# Supplementary material for: Ancestral origins and invasion pathways in a globally invasive bird correlate with climate and influences from bird trade
Source: Mol Ecol. 2015 Aug 3;24(16):4269–85. doi: 10.1111/mec.13307 (PMC4657503; doi:10.1111/mec.13307)
Supplement: Supplementary file 1 [file mec0024-4269-sd1.docx]

Jackson *et al.* – Supplementary Material

Ancestral origins and invasion pathways of a globally invasive bird correlate with climate and influences from bird trade*.*

**Table S1**: Sample size and location information for invasive populations included in this study. N-msat = number of individuals genotyped at 10 microsatellite loci; N-mtDNA=number of individuals sequenced for two mitochondrial markers.

| Population | N | N-msat | N-mtDNA | Lat | Long |
| --- | --- | --- | --- | --- | --- |
| Amsterdam | 19 | 17 | 19 | 52.37 | 4.89 |
| Bonn | 29 | 29 | 29 | 50.73 | 7.10 |
| Brussels | 69 | 69 | 65 | 50.84 | 4.36 |
| Dusseldorf | 9 | 9 | 9 | 51.23 | 6.78 |
| Greater London | 195 | 163 | 195 | 51.52 | 0.11 |
| Heidelberg | 185 | 185 | 95 | 49.40 | 8.68 |
| Madrid | 2 | 1 | 2 | 40.40 | -3.68 |
| Marseille | 2 | 2 | 2 | 43.30 | 5.38 |
| Mauritius | 115 | 115 | 52 | -20.16 | 57.50 |
| Rotterdam | 75 | 75 | 74 | 51.92 | 4.48 |
| Seville | 57 | 57 | 56 | 37.38 | -5.98 |
| Seychelles | 2 | 2 | 2 | -4.63 | 55.46 |
| The Hague | 13 | 12 | 13 | 52.08 | 4.311 |
| Tuscany | 1 | 1 | 1 | 43.41 | 11.00 |
| Utrecht | 2 | 2 | 2 | 52.09 | 5.12 |
| Wiesbaden | 80 | 80 | 80 | 50.09 | 8.24 |

**Table S2**: Sample and location information for historical specimens from the Natural History Museum in Tring, UK

| Subspp. designation | Museum ID | Sample location | Lat | Long |
| --- | --- | --- | --- | --- |
| *P. k. borealis* | 1889.1.26.271 | Assam | 27.83 | 95.67 |
| *P. k. borealis* | 1889.1.26.258 | Bhutan | 26.74 | 89.74 |
| *P. k. borealis* | 1889.1.26.288 | Bhutan | 26.74 | 89.74 |
| *P. k. borealis* | 1941.5.30.2913 | Burma | 18.33 | 95.62 |
| *P. k. borealis* | 1908.5.30.84 | Burma | 18.33 | 95.62 |
| *P. k. borealis* | 1948.80.3671 | Burma Myanmar | 18.33 | 95.62 |
| *P. k. borealis* | 1889.1.26.289 | Calcutta Bhutan | 22.56 | 88.36 |
| *P. k. borealis* | 1889.1.26.290 | Calcutta India | 22.56 | 88.36 |
| *P. k. borealis* | 1889.1.26.286 | Dacca Bangladesh | 23.70 | 90.37 |
| *P. k. borealis* | 1889.1.26.254 | Darjeeling India | 27.03 | 88.16 |
| *P. k. borealis* | 1889.1.26.245 | Dehli India | 28.46 | 77.03 |
| *P. k. borealis* | 1889.1.26.273 | Dibrugarh India | 27.48 | 94.99 |
| *P. k. borealis* | 1889.1.26.291 | Dibrughur India | 27.48 | 94.99 |
| *P. k. borealis* | 1889.1.26.252 | Etawah India | 26.77 | 79.03 |
| *P. k. borealis* | 1889.1.26.253 | Etawah India | 26.77 | 79.03 |
| *P. k. borealis* | 1889.1.26.237 | Jhelum Pakistan | 32.92 | 73.73 |
| *P. k. borealis* | 1875.7.13.72 | Kamrtee India | 21.23 | 79.20 |
| *P. k. borealis* | 1875.7.13.73 | Kamthi India | 21.23 | 79.20 |
| *P. k. borealis* | 1889.1.20.360 | Lawrencepur Pakistan | 33.83 | 72.50 |
| *P. k. borealis* | 1889.1.20.355 | Maunbhoom India | 19.08 | 74.72 |
| *P. k. borealis* | 1897.12.10.1842 | Meerut India | 28.99 | 77.7 |
| *P. k. borealis* | 1884.10.8.50 | Mhow India | 22.55 | 75.76 |
| *P. k. borealis* | 1889.1.26.256 | Nepal | 13.47 | 5.87 |
| *P. k. borealis* | 1949.Whi.1.16880 | Punjab | 30.16 | 76.87 |
| *P. k. borealis* | 1949.25.853 | Punjab | 32.71 | 72.98 |
| *P. k. borealis* | 1949.25.854 | Punjab India | 30.20 | 70.72 |
| *P. k. borealis* | 1949.25.856 | Punjab India | 30.35 | 71.39 |
| *P. k. borealis* | 1949.Whi.1.16883 | Punjab Pakistan | 30.20 | 71.41 |
| *P. k. borealis* | 1881.5.1.4725 | Punjab Pakistan | 32.54 | 71.93 |
| *P. k. borealis* | 1949.25.850 | Punjab Pakistan | 32.08 | 72.67 |
| *P. k. borealis* | 1949.25.851 | Punjab Pakistan | 32.78 | 72.70 |
| *P. k. borealis* | 1949.25.855 | Punjab Pakistan | 26.30 | 74.73 |
| *P. k. borealis* | 1949.Whi.1.16956 | Rawalpindi Pakistan | 11.83 | 32.80 |
| *P. k. borealis* | 1889.1.26.240 | Sambhal India | 14.83 | -17.10 |
| *P. k. borealis* | 1889.1.26.278 | Seoni India | 28.00 | 68.40 |
| *P. k. borealis* | 1860.4.16.550 | Shikarpur Pakistan | 26.42 | 67.86 |
| *P. k. borealis* | 1898.12.12.320 | Sind India | 27.68 | 68.86 |
| *P. k. borealis* | 1889.1.20.235 | Sindh Punjab | 28.46 | 77.03 |
| *P. k. borealis* | 1889.1.26.246 | Sindh Punjab | 32.30 | 75.90 |
| *P. k. borealis* | 1889.1.26.248 | Sindh Punjab | 30.38 | 76.78 |
| *P. k. borealis* | 1889.1.20.358 | Sindh Punjab | 25.37 | 68.36 |
| *P. k. borealis* | 1860.4.16.557 | Sindh Punjab | 14.61 | 74.83 |
| *P. k. borealis* | 1889.1.26.262 | Suddya Bhutan | 27.68 | 68.86 |
| *P. k. borealis* | 1889.1.26.263 | Suddya India | 27.68 | 68.86 |
| *P. k. borealis* | 1889.1.26.236 | Sukkur Pakistan | 14.83 | -17.10 |
| *P. k. krameri* | 1915.12.24.510 | Bahr el Ghazal Sudan | 7.70 | 28.00 |
| *P. k. krameri* | 1907.12.23.77 | Bahr el Ghazal Sudan | 7.70 | 28.00 |
| *P. k. krameri* | 1929.2.18.122 | Bakalari Gambia | 13.42 | -16.42 |
| *P. k. krameri* | 1923.10.26.67 | Cameroon | 9.30 | 13.40 |
| *P. k. krameri* | 1920.12.22.105 | Dafur Sudan | 11.07 | 26.85 |
| *P. k. krameri* | 1922.12.8.406 | Dafur Sudan | 12.90 | 23.48 |
| *P. k. krameri* | 1922.12.8.408 | Dafur Sudan | 12.59 | 23.61 |
| *P. k. krameri* | 1922.12.8.410 | Dafur Sudan | 12.90 | 23.48 |
| *P. k. krameri* | 1922.12.8.412 | Dafur Sudan | 15.13 | 26.17 |
| *P. k. krameri* | 1922.12.8.414 | Dafur Sudan | 13.63 | 25.35 |
| *P. k. krameri* | 1929.2.18.121 | Gambia | 13.42 | -16.42 |
| *P. k. krameri* | 1899.9.20.5 | Ghana | 10.50 | -1.96 |
| *P. k. krameri* | 1910.5.6.154 | Guinea | 12.31 | -15.78 |
| *P. k. krameri* | 1910.5.6.153 | Gunnal Guinea Bissau | 12.31 | -15.78 |
| *P. k. krameri* | 1930.3.4.371 | Haute Volta | 12.13 | 0.65 |
| *P. k. krameri* | 1939.12.9.3243 | Kael Senegal | 14.70 | -15.89 |
| *P. k. krameri* | 1929.2.18.120 | Kerewan Gambia | 13.50 | -16.08 |
| *P. k. krameri* | 1922.12.8.405 | Kurdufan Sudan | 13.18 | 30.21 |
| *P. k. krameri* | 1926.8.8.136 | LakeChad Nigeria | 14.25 | 13.11 |
| *P. k. krameri* | 1915.12.24.512 | Mongalla Sudan | 5.19 | 31.76 |
| *P. k. krameri* | 1928.7.20.26 | Nigeria | 9.153 | 4.812 |
| *P. k. krameri* | 1900.8.4.29 | Nigeria | 13.55 | 13.23 |
| *P. k. krameri* | 1911.12.23.535 | Nigeria | 18.52 | 73.85 |
| *P. k. krameri* | 1923.8.7.7000 | Renk Sudan | 11.83 | 32.80 |
| *P. k. krameri* | 1923.8.7.7001 | Renk Sudan | 28.58 | 78.55 |
| *P. k. krameri* | 1939.12.9.3241 | Senegal Gambia | 14.66 | -16.25 |
| *P. k. krameri* | 1918.8.26.33 | Senegal Gambia | 16.03 | -16.50 |
| *P. k. krameri* | 1889.1.20.331 | Senegal Gambia | 22.08 | 79.53 |
| *P. k. krameri* | 1907.12.23.76 | Sudan | 26.74 | 89.74 |
| *P. k. krameri* | 1939.12.9.3240 | Thies Senegal | 8.66 | 76.76 |
| *P. k. krameri* | 1911.12.23.536 | Yo Nigeria | 27.83 | 95.67 |
| *P. k. manillensis* | 1889.1.26.283 | Aujango India | 8.66 | 76.76 |
| *P. k. manillensis* | 1940.12.3.211 | Kalawewa Sri Lanka | 8.01 | 80.51 |
| *P. k. manillensis* | 1949.Whi.1.16889 | Madras India | 9.74 | 77.30 |
| *P. k. manillensis* | 1884.7.28.38 | Mysore India | 12.30 | 76.65 |
| *P. k. manillensis* | 1860.4.16.558 | Mysore India | 12.30 | 76.65 |
| *P. k. manillensis* | 1919.1.12.61 | Pune India | 30.35 | 71.39 |
| *P. k. manillensis* | 1949.Whi.1.16884 | Rajasthan India | 33.60 | 73.03 |
| *P. k. manillensis* | 1925.12.23.1106 | Sirsi Kanara | 8.01 | 80.51 |
| *P. k. manillensis* | 1940.12.3.209 | Sri Lanka | 6.12 | 81.12 |
| *P. k. manillensis* | 1946.28.234 | Sri Lanka | 6.83 | 79.87 |
| *P. k. manillensis* | 1953.16.31 | Sri Lanka | 8.70 | 25.45 |
| *P. k. manillensis* | 1937.12.21.94 | Travancore | 13.55 | 13.23 |
| *P. k. manillensis* | 1889.1.26.281 | Travancore India | 15.78 | 38.45 |
| *P. k. parvirostris* | 1890.10.10.4 | Anseba River Ethiopia | 15.78 | 38.45 |
| *P. k. parvirostris* | 1927.5.3.1 | Berbera Somalia | 10.43 | 45.01 |
| *P. k. parvirostris* | 1915.12.24.515 | BlueNile Sudan | 11.85 | 34.38 |
| *P. k. parvirostris* | 1889.1.20.334 | Eritrea Africa | 15.90 | 38.45 |
| *P. k. parvirostris* | 1878.12.31.663 | Eritrea Africa | 15.90 | 38.45 |
| *P. k. parvirostris* | 1919.12.17.751 | Kamisa Sudan | 13.12 | 34.22 |
| *P. k. parvirostris* | 1915.12.24.513 | Mongalla Sudan | 5.19 | 31.76 |

**Table S3:** Latitudes for native range Asian haplotypes and their frequency in invasive European populations.

| Native Asian haplotype number | No of individuals in each haplotype | Latitude (min) | Latitude (max) | Latitude (mean) | Frequency in invasive European populations |
| --- | --- | --- | --- | --- | --- |
| 4 | 3 | 6.12 | 25.38 | 18.02 | 11 |
| 5 | 6 | 27.68 | 30.35 | 29.56 | 153 |
| 7 | 3 | 14.62 | 26.77 | 19.97 | 1 |
| 8 | 3 | 10.81 | 32.78 | 25.30 | 17 |
| 9 | 1 | 28.00 | 28.00 | 28.00 | 1 |
| 18 | 16 | 22.57 | 32.93 | 27.80 | 366 |
| 19 | 1 | 33.60 | 33.60 | 33.60 | 0 |
| 20 | 2 | 26.75 | 33.83 | 30.29 | 4 |
| 21 | 1 | 28.99 | 28.99 | 28.99 | 0 |
| 22 | 1 | 32.72 | 32.72 | 32.72 | 0 |
| 23 | 1 | 27.68 | 27.68 | 27.68 | 1 |
| 24 | 6 | 8.66 | 26.43 | 16.56 | 34 |
| 25 | 1 | 19.08 | 19.08 | 19.08 | 0 |
| 26 | 1 | 12.30 | 12.30 | 12.30 | 0 |
| 27 | 1 | 21.23 | 21.23 | 21.23 | 0 |
| 28 | 1 | 26.30 | 26.30 | 26.30 | 10 |
| 29 | 1 | 8.02 | 8.02 | 8.02 | 0 |
| 30 | 1 | 6.84 | 6.84 | 6.84 | 0 |
| 31 | 1 | 9.74 | 9.74 | 9.74 | 0 |
| 33 | 1 | 32.54 | 32.54 | 32.54 | 1 |
| 34 | 1 | 27.68 | 27.68 | 27.68 | 0 |
| 35 | 1 | 23.70 | 23.70 | 23.70 | 0 |
| 36 | 2 | 0.00 | 18.34 | 9.17 | 0 |
| 37 | 1 | 18.34 | 18.34 | 18.34 | 0 |
| 38 | 1 | 18.34 | 18.34 | 18.34 | 0 |

**Table S4**. Haplotype frequencies for each of the 74 mtDNA haplotypes discovered across the native and invasive ranges.

| **Pop/Hap No** | **1** | **2** | **3** | **4** | **5** | **6** | **7** | **8** | **9** | **10** | **11** | **12** | **13** | **14** | **15** | **16** | **17** | **18** | **19** | **20** | **21** | **22** | **23** | **24** | **25** |
| --- | --- | --- | --- | --- | --- | --- | --- | --- | --- | --- | --- | --- | --- | --- | --- | --- | --- | --- | --- | --- | --- | --- | --- | --- | --- |
| Bonn | 0 | 0 | 0 | 0 | 2 | 0 | 0 | 0 | 0 | 0 | 0 | 0 | 0 | 0 | 0 | 0 | 0 | 11 | 0 | 0 | 0 | 0 | 0 | 2 | 0 |
| Brussels | 0 | 0 | 0 | 0 | 8 | 0 | 0 | 1 | 0 | 0 | 0 | 0 | 0 | 0 | 0 | 0 | 0 | 35 | 0 | 0 | 0 | 0 | 0 | 2 | 0 |
| Dusseldorf | 0 | 0 | 0 | 0 | 0 | 0 | 0 | 0 | 0 | 0 | 0 | 0 | 0 | 0 | 0 | 0 | 0 | 0 | 0 | 4 | 0 | 0 | 0 | 0 | 0 |
| Marseille | 0 | 0 | 0 | 0 | 2 | 0 | 0 | 0 | 0 | 0 | 0 | 0 | 0 | 0 | 0 | 0 | 0 | 0 | 0 | 0 | 0 | 0 | 0 | 0 | 0 |
| Seychelles | 0 | 0 | 0 | 0 | 0 | 0 | 0 | 0 | 0 | 0 | 0 | 0 | 0 | 0 | 0 | 0 | 0 | 2 | 0 | 0 | 0 | 0 | 0 | 0 | 0 |
| Tuscany | 0 | 0 | 0 | 0 | 0 | 0 | 0 | 0 | 0 | 0 | 0 | 0 | 0 | 0 | 0 | 0 | 0 | 0 | 0 | 0 | 0 | 0 | 0 | 0 | 0 |
| Mauritius | 0 | 0 | 0 | 4 | 44 | 0 | 0 | 0 | 0 | 0 | 0 | 0 | 0 | 0 | 0 | 0 | 0 | 2 | 0 | 0 | 0 | 0 | 0 | 0 | 0 |
| Heidelberg | 0 | 0 | 1 | 0 | 2 | 0 | 0 | 0 | 0 | 0 | 0 | 0 | 0 | 0 | 0 | 0 | 0 | 91 | 0 | 0 | 0 | 0 | 0 | 0 | 0 |
| Madrid | 0 | 0 | 0 | 0 | 2 | 0 | 0 | 0 | 0 | 0 | 0 | 0 | 0 | 0 | 0 | 0 | 0 | 0 | 0 | 0 | 0 | 0 | 0 | 0 | 0 |
| Seville | 0 | 0 | 0 | 1 | 54 | 0 | 0 | 0 | 0 | 0 | 0 | 0 | 0 | 0 | 0 | 0 | 0 | 0 | 0 | 0 | 0 | 0 | 0 | 1 | 0 |
| Greater London | 1 | 0 | 2 | 9 | 29 | 0 | 1 | 0 | 1 | 0 | 0 | 0 | 0 | 0 | 0 | 0 | 0 | 112 | 0 | 0 | 0 | 0 | 1 | 8 | 0 |
| Wiesbaden | 0 | 0 | 0 | 1 | 4 | 0 | 0 | 3 | 0 | 0 | 0 | 0 | 0 | 0 | 0 | 0 | 0 | 54 | 0 | 0 | 0 | 0 | 0 | 10 | 0 |
| Rotterdam | 0 | 0 | 0 | 2 | 7 | 0 | 0 | 11 | 0 | 0 | 0 | 0 | 0 | 0 | 0 | 0 | 0 | 38 | 0 | 0 | 0 | 0 | 0 | 10 | 0 |
| The Hague | 0 | 0 | 0 | 2 | 3 | 0 | 0 | 0 | 0 | 0 | 0 | 0 | 0 | 0 | 0 | 0 | 0 | 6 | 0 | 0 | 0 | 0 | 0 | 2 | 0 |
| Amsterdam | 0 | 0 | 0 | 0 | 2 | 0 | 0 | 0 | 0 | 0 | 0 | 0 | 0 | 0 | 0 | 0 | 0 | 16 | 0 | 0 | 0 | 0 | 0 | 1 | 0 |
| Utrecht | 0 | 0 | 0 | 0 | 0 | 0 | 0 | 0 | 0 | 0 | 0 | 0 | 0 | 0 | 0 | 0 | 0 | 2 | 0 | 0 | 0 | 0 | 0 | 0 | 0 |
| Asia | 0 | 0 | 0 | 3 | 6 | 0 | 3 | 3 | 1 | 0 | 0 | 0 | 0 | 0 | 0 | 0 | 0 | 16 | 1 | 2 | 1 | 1 | 1 | 6 | 1 |
| Africa | 1 | 13 | 1 | 0 | 0 | 1 | 0 | 0 | 0 | 1 | 1 | 3 | 1 | 1 | 4 | 1 | 1 | 0 | 0 | 0 | 0 | 0 | 0 | 0 | 0 |

**Table S4 cont.**

| **Pop/Hap No** | **26** | **27** | **28** | **29** | **30** | **31** | **32** | **33** | **34** | **35** | **36** | **37** | **38** | **39** | **40** | **41** | **42** | **43** | **44** | **45** | **46** | **47** | **48** | **49** | **50** |
| --- | --- | --- | --- | --- | --- | --- | --- | --- | --- | --- | --- | --- | --- | --- | --- | --- | --- | --- | --- | --- | --- | --- | --- | --- | --- |
| Bonn | 0 | 0 | 0 | 0 | 0 | 0 | 0 | 0 | 0 | 0 | 0 | 0 | 0 | 0 | 0 | 0 | 0 | 0 | 0 | 0 | 1 | 0 | 4 | 1 | 0 |
| Brussels | 0 | 0 | 0 | 0 | 0 | 0 | 0 | 0 | 0 | 0 | 0 | 0 | 0 | 0 | 0 | 0 | 0 | 0 | 0 | 0 | 0 | 0 | 0 | 0 | 0 |
| Dusseldorf | 0 | 0 | 0 | 0 | 0 | 0 | 0 | 0 | 0 | 0 | 0 | 0 | 0 | 0 | 0 | 0 | 0 | 0 | 0 | 0 | 0 | 0 | 0 | 0 | 0 |
| Marseille | 0 | 0 | 0 | 0 | 0 | 0 | 0 | 0 | 0 | 0 | 0 | 0 | 0 | 0 | 0 | 0 | 0 | 0 | 0 | 0 | 0 | 0 | 0 | 0 | 0 |
| Seychelles | 0 | 0 | 0 | 0 | 0 | 0 | 0 | 0 | 0 | 0 | 0 | 0 | 0 | 0 | 0 | 0 | 0 | 0 | 0 | 0 | 0 | 0 | 0 | 0 | 0 |
| Tuscany | 0 | 0 | 0 | 0 | 0 | 0 | 0 | 1 | 0 | 0 | 0 | 0 | 0 | 0 | 0 | 0 | 0 | 0 | 0 | 0 | 0 | 0 | 0 | 0 | 0 |
| Mauritius | 0 | 0 | 0 | 0 | 0 | 0 | 0 | 0 | 0 | 0 | 0 | 0 | 0 | 0 | 0 | 0 | 0 | 0 | 0 | 0 | 0 | 0 | 0 | 0 | 0 |
| Heidelberg | 0 | 0 | 0 | 0 | 0 | 0 | 0 | 0 | 0 | 0 | 0 | 0 | 0 | 0 | 0 | 0 | 0 | 0 | 0 | 0 | 0 | 0 | 1 | 0 | 0 |
| Madrid | 0 | 0 | 0 | 0 | 0 | 0 | 0 | 0 | 0 | 0 | 0 | 0 | 0 | 0 | 0 | 0 | 0 | 0 | 0 | 0 | 0 | 0 | 0 | 0 | 0 |
| Seville | 0 | 0 | 0 | 0 | 0 | 0 | 0 | 0 | 0 | 0 | 0 | 0 | 0 | 0 | 0 | 0 | 0 | 0 | 0 | 0 | 0 | 0 | 1 | 0 | 0 |
| Greater London | 0 | 0 | 10 | 0 | 0 | 0 | 1 | 0 | 0 | 0 | 0 | 0 | 0 | 0 | 0 | 0 | 0 | 0 | 0 | 0 | 0 | 0 | 12 | 0 | 0 |
| Wiesbaden | 0 | 0 | 0 | 0 | 0 | 0 | 0 | 0 | 0 | 0 | 0 | 0 | 0 | 0 | 0 | 0 | 0 | 0 | 0 | 0 | 1 | 0 | 0 | 1 | 0 |
| Rotterdam | 0 | 0 | 0 | 0 | 0 | 0 | 0 | 0 | 0 | 0 | 0 | 0 | 0 | 0 | 0 | 0 | 0 | 0 | 0 | 1 | 1 | 1 | 1 | 1 | 1 |
| The Hague | 0 | 0 | 0 | 0 | 0 | 0 | 0 | 0 | 0 | 0 | 0 | 0 | 0 | 0 | 0 | 0 | 0 | 0 | 0 | 0 | 0 | 0 | 0 | 0 | 0 |
| Amsterdam | 0 | 0 | 0 | 0 | 0 | 0 | 0 | 0 | 0 | 0 | 0 | 0 | 0 | 0 | 0 | 0 | 0 | 0 | 0 | 0 | 0 | 0 | 0 | 0 | 0 |
| Utrecht | 0 | 0 | 0 | 0 | 0 | 0 | 0 | 0 | 0 | 0 | 0 | 0 | 0 | 0 | 0 | 0 | 0 | 0 | 0 | 0 | 0 | 0 | 0 | 0 | 0 |
| Asia | 1 | 1 | 1 | 1 | 1 | 1 | 1 | 1 | 1 | 1 | 1 | 1 | 1 | 0 | 0 | 0 | 0 | 0 | 0 | 0 | 0 | 0 | 0 | 0 | 0 |
| Africa | 0 | 0 | 0 | 0 | 0 | 0 | 0 | 0 | 0 | 0 | 0 | 0 | 0 | 1 | 1 | 1 | 1 | 4 | 1 | 0 | 0 | 0 | 0 | 0 | 0 |

**Table S4 cont.**

| **Pop/Hap No** | **51** | **52** | **53** | **54** | **55** | **56** | **57** | **58** | **59** | **60** | **61** | **62** | **63** | **64** | **65** | **66** | **67** | **68** | **69** | **70** | **71** | **72** | **73** | **74** |
| --- | --- | --- | --- | --- | --- | --- | --- | --- | --- | --- | --- | --- | --- | --- | --- | --- | --- | --- | --- | --- | --- | --- | --- | --- |
| Bonn | 4 | 0 | 0 | 0 | 0 | 0 | 1 | 0 | 0 | 0 | 0 | 0 | 0 | 0 | 1 | 1 | 0 | 0 | 0 | 0 | 0 | 0 | 0 | 1 |
| Brussels | 0 | **9** | 0 | 0 | 0 | 0 | 0 | 8 | 0 | 0 | 0 | 0 | 0 | 0 | 0 | 0 | 1 | 1 | 0 | 0 | 0 | 0 | 0 | 0 |
| Dusseldorf | 0 | 0 | 0 | 1 | 0 | 3 | 0 | 0 | 0 | 0 | 0 | 0 | 0 | 0 | 0 | 0 | 0 | 0 | 0 | 0 | 0 | 1 | 0 | 0 |
| Marseille | 0 | 0 | 0 | 0 | 0 | 0 | 0 | 0 | 0 | 0 | 0 | 0 | 0 | 0 | 0 | 0 | 0 | 0 | 0 | 0 | 0 | 0 | 0 | 0 |
| Seychelles | 0 | 0 | 0 | 0 | 0 | 0 | 0 | 0 | 0 | 0 | 0 | 0 | 0 | 0 | 0 | 0 | 0 | 0 | 0 | 0 | 0 | 0 | 0 | 0 |
| Tuscany | 0 | 0 | 0 | 0 | 0 | 0 | 0 | 0 | 0 | 0 | 0 | 0 | 0 | 0 | 0 | 0 | 0 | 0 | 0 | 0 | 0 | 0 | 0 | 0 |
| Mauritius | 0 | 0 | 0 | 0 | 0 | 0 | 0 | 0 | 0 | 0 | 0 | 0 | 0 | 0 | 0 | 0 | 0 | 0 | 0 | 0 | 0 | 0 | 2 | 0 |
| Heidelberg | 0 | 0 | 0 | 0 | 0 | 0 | 0 | 0 | 0 | 0 | 0 | 0 | 0 | 0 | 0 | 0 | 0 | 0 | 0 | 0 | 0 | 0 | 0 | 0 |
| Madrid | 0 | 0 | 0 | 0 | 0 | 0 | 0 | 0 | 0 | 0 | 0 | 0 | 0 | 0 | 0 | 0 | 0 | 0 | 0 | 0 | 0 | 0 | 0 | 0 |
| Seville | 0 | 0 | 0 | 0 | 0 | 0 | 0 | 0 | 0 | 0 | 0 | 0 | 0 | 0 | 0 | 0 | 0 | 0 | 1 | 0 | 0 | 0 | 0 | 0 |
| Greater London | 0 | 0 | 2 | 0 | 1 | 0 | 1 | 3 | 1 | 2 | 0 | 0 | 0 | 0 | 0 | 0 | 0 | 0 | 0 | 0 | 0 | 0 | 0 | 0 |
| Wiesbaden | 0 | 0 | 0 | 0 | 0 | 0 | 0 | 0 | 0 | 0 | 1 | 1 | 1 | 1 | 0 | 0 | 0 | 0 | 0 | 1 | 1 | 0 | 0 | 0 |
| Rotterdam | 0 | 0 | 0 | 0 | 0 | 0 | 0 | 0 | 0 | 0 | 0 | 0 | 0 | 0 | 0 | 0 | 0 | 0 | 0 | 0 | 0 | 0 | 0 | 0 |
| The Hague | 0 | 0 | 0 | 0 | 0 | 0 | 0 | 0 | 0 | 0 | 0 | 0 | 0 | 0 | 0 | 0 | 0 | 0 | 0 | 0 | 0 | 0 | 0 | 0 |
| Amsterdam | 0 | 0 | 0 | 0 | 0 | 0 | 0 | 0 | 0 | 0 | 0 | 0 | 0 | 0 | 0 | 0 | 0 | 0 | 0 | 0 | 0 | 0 | 0 | 0 |
| Utrecht | 0 | 0 | 0 | 0 | 0 | 0 | 0 | 0 | 0 | 0 | 0 | 0 | 0 | 0 | 0 | 0 | 0 | 0 | 0 | 0 | 0 | 0 | 0 | 0 |
| Asia | 0 | 0 | 0 | 0 | 0 | 0 | 0 | 0 | 0 | 0 | 0 | 0 | 0 | 0 | 0 | 0 | 0 | 0 | 0 | 0 | 0 | 0 | 0 | 0 |
| Africa | 0 | 0 | 0 | 0 | 0 | 0 | 0 | 0 | 0 | 0 | 0 | 0 | 0 | 0 | 0 | 0 | 0 | 0 | 0 | 0 | 0 | 0 | 0 | 0 |

**Table S5**. Model checking for scenario 3 in DIYABC based upon 10^6^ simulated datasets. For each summary statistic we calculated the proportion of datasets in which the statistic was less than the observed value. Values <5% or >95% (*) are indicative of a poor match; O=observed, P=proportion of simulated<observed. One and two sample summary statistics comprise; NAL & N2P: mean number of alleles, HET & H2P: gene diversity, VAR & V2P: mean size variance, DTA: Tajima’s J and HST: F_ST._

|  | **Brussels** | | **Heidelberg** | | **Wiesbaden** | | **Bonn** | | **Dusseldorf** | | **Seville** | |
| --- | --- | --- | --- | --- | --- | --- | --- | --- | --- | --- | --- | --- |
| **Summary statistics** | **O** | **P** | **O** | **P** | **O** | **P** | **O** | **P** | **O** | **P** | **O** | **P** |
| NAL_1_1 | 9.4444 | 0.15 | 8 | 0.1795 | 8 | 0.1305 | 7.7778 | 0.344 | 6.1111 | 0.4455 | 9.5556 | 0.2075 |
| NAL_1_2 | 7 | 0.5405 | 7 | 0.5185 | 7 | 0.5315 | 7 | 0.482 | 7 | 0.585 | 7 | 0.526 |
| NAL_1_3 | 6 | 0.5855 | 6 | 0.5195 | 6 | 0.614 | 6 | 0.365 | 5.8889 | 0.394 | 6 | 0.5175 |
| HET_1_1 | 0.7761 | 0.168 | 0.7395 | 0.1465 | 0.7785 | 0.236 | 0.777 | 0.285 | 0.8223 | 0.587 | 0.7739 | 0.1345 |
| HET_1_2 | 0.7014 | 0.31 | 0.7014 | 0.3355 | 0.7014 | 0.3045 | 0.7014 | 0.278 | 0.7014 | 0.3595 | 0.7014 | 0.3055 |
| HET_1_3 | 0.6654 | 0.2835 | 0.6654 | 0.2685 | 0.6654 | 0.3245 | 0.6654 | 0.1405 | 0.6631 | 0.173 | 0.6654 | 0.253 |
| VAR_1_1 | 13.1456 | 0.054 | 13.1787 | 0.305 | 12.7845 | 0.029* | 15.2729 | 0.056 | 15.6542 | 0.058 | 13.2793 | 0.032* |
| VAR_1_2 | 7.2802 | 0.638 | 7.2802 | 0.552 | 7.2802 | 0.579 | 7.2802 | 0.567 | 7.2802 | 0.675 | 7.2802 | 0.5965 |
| VAR_1_3 | 7.5445 | 0.742 | 7.5445 | 0.631 | 7.5445 | 0.74 | 7.5445 | 0.592 | 7.5398 | 0.6455 | 7.5445 | 0.69 |
| N2P_1_1&2 | 10.2222 | 0.1605 | 9.7778 | 0.246 | 9.5556 | 0.134 | 9.6667 | 0.222 | 8.7778 | 0.2565 | 10.5556 | 0.196 |
| N2P_1_1&3 | 10.4444 | 0.1595 | 9.7778 | 0.275 | 9.4444 | 0.162 | 9.5556 | 0.2095 | 8.4444 | 0.3085 | 10.7778 | 0.195 |
| H2P_1_1&2 | 0.7918 | 0.349 | 0.76 | 0.2115 | 0.7971 | 0.383 | 0.8039 | 0.5125 | 0.807 | 0.656 | 0.798 | 0.3865 |
| H2P_1_1&3 | 0.7871 | 0.142 | 0.7533 | 0.1755 | 0.7945 | 0.2805 | 0.804 | 0.358 | 0.8006 | 0.5585 | 0.7906 | 0.16 |
| V2P_1_1&2 | 11.9167 | 0.062 | 12.7685 | 0.298 | 11.5661 | 0.028* | 11.7526 | 0.052 | 10.8215 | 0.079 | 12.2073 | 0.041* |
| V2P_1_1&3 | 12.3919 | 0.0235* | 13.0729 | 0.29 | 12.0812 | 0.022* | 12.6675 | 0.034* | 11.7301 | 0.109 | 12.7567 | 0.018* |
| DTA_2_1 | -1.2407 | 0.016* | -1.2312 | 0.006** | 0.6816 | 0.194 | -0.1559 | 0.083 | 0.4286 | 0.501 | -1.1996 | 0.016* |
| DTA_2_2 | 0.4732 | 0.8385 | 0.5902 | 0.891 | 0.5902 | 0.923 | 0.5902 | 0.933 | -0.7193 | 0.0845 | 0.5902 | 0.877 |
| DTA_2_3 | 0.2217 | 0.7945 | 0.3793 | 0.842 | 0.3793 | 0.908 | 0.3793 | 0.911 | -0.1693 | 0.145 | 0.3793 | 0.841 |
| HST_2_1&2 | 0.4864 | 0.8795 | 0.4172 | 0.765 | 0.1 | 0.3925 | 0.22 | 0.139 | -0.1951 | 0.006** | 0.2822 | 0.606 |
| HST_2_1&3 | 0.6095 | 0.68 | 0.4591 | 0.7795 | 0.1273 | 0.2865 | 0.2512 | 0.11 | 0.1738 | 0.132 | 0.3351 | 0.31 |

**Table S5 cont.**

|  | **Greater London** | | **Rotterdam** | | **The Hague** | | **Amsterdam** | | **Mauritius** | |
| --- | --- | --- | --- | --- | --- | --- | --- | --- | --- | --- |
| **Summary statistics** | **O** | **P** | **O** | **P** | **O** | **P** | **O** | **P** | **O** | **P** |
| NAL_1_1 | 10.7778 | 0.183 | 9.4444 | 0.257 | 5.3333 | 0.0615 | 6.8889 | 0.0905 | 7.1111 | 0.135 |
| NAL_1_2 | 7 | 0.5955 | 7 | 0.5065 | 7 | 0.6 | 7 | 0.4905 | 7 | 0.417 |
| NAL_1_3 | 6 | 0.523 | 6 | 0.482 | 6 | 0.4395 | 6 | 0.419 | 6 | 0.403 |
| HET_1_1 | 0.8044 | 0.268 | 0.7667 | 0.1015 | 0.6925 | 0.0375* | 0.7786 | 0.1655 | 0.6915 | 0.1045 |
| HET_1_2 | 0.7014 | 0.3585 | 0.7014 | 0.2785 | 0.7014 | 0.3485 | 0.7014 | 0.286 | 0.7014 | 0.309 |
| HET_1_3 | 0.6654 | 0.267 | 0.6654 | 0.224 | 0.6654 | 0.19 | 0.6654 | 0.169 | 0.6654 | 0.223 |
| VAR_1_1 | 17.0642 | 0.066 | 14.1607 | 0.037* | 12.9551 | 0.052 | 18.1046 | 0.083 | 7.941 | 0.001*** |
| VAR_1_2 | 7.2802 | 0.6345 | 7.2802 | 0.609 | 7.2802 | 0.6375 | 7.2802 | 0.565 | 3.7039 | 0.1895 |
| VAR_1_3 | 7.5445 | 0.669 | 7.5445 | 0.687 | 7.5445 | 0.664 | 7.5445 | 0.645 | 4.0529 | 0.269 |
| N2P_1_1&2 | 11.4444 | 0.173 | 10.4444 | 0.2005 | 8.5556 | 0.1765 | 9.2222 | 0.1585 | 12.5556 | 0.383 |
| N2P_1_1&3 | 11.5556 | 0.193 | 10.3333 | 0.185 | 8 | 0.1265 | 9 | 0.113 | 11.7778 | 0.351 |
| H2P_1_1&2 | 0.8098 | 0.322 | 0.7911 | 0.2595 | 0.791 | 0.554 | 0.7995 | 0.504 | 0.7892 | 0.294 |
| H2P_1_1&3 | 0.8068 | 0.2395 | 0.7814 | 0.105 | 0.7821 | 0.3695 | 0.7941 | 0.271 | 0.7662 | 0.2085 |
| V2P_1_1&2 | 16.0579 | 0.06 | 13.1946 | 0.043* | 10.9422 | 0.111 | 12.8484 | 0.099 | 35.0119 | 0.174 |
| V2P_1_1&3 | 16.5585 | 0.049* | 13.7699 | 0.028* | 12.3257 | 0.093 | 14.4765 | 0.058 | 28.8592 | 0.099 |
| DTA_2_1 | -1.3783 | 0.005** | -0.7571 | 0.032* | 0.0823 | 0.413 | -0.899 | 0.033* | -0.2847 | 0.085 |
| DTA_2_2 | 0.3966 | 0.8475 | 0.5902 | 0.881 | 0.3966 | 0.883 | 0.5902 | 0.941 | 0.3966 | 0.861 |
| DTA_2_3 | 0.3793 | 0.8405 | 0.3793 | 0.825 | 0.2217 | 0.858 | 0.3793 | 0.919 | 0.2217 | 0.858 |
| HST_2_1&2 | 0.5472 | 0.954* | 0.2578 | 0.5935 | 0.0784 | 0.125 | 0.1352 | 0.142 | 0.3726 | 0.063 |
| HST_2_1&3 | 0.5967 | 0.8965 | 0.2953 | 0.408 | 0.0159 | 0.109 | 0.1079 | 0.114 | 0.4075 | 0.224 |

**Table S6**. Measures of performance; mean bias and RRMISE in support of scenario three in DIYABC based upon 10^6^ simulated datasets.

| **Priors** | **Brussels** | | **Heidelberg** | | **Wiesbaden** | | **Bonn** | | **Dusseldorf** | |
| --- | --- | --- | --- | --- | --- | --- | --- | --- | --- | --- |
|  | **Bias** | **RRMISE** | **Bias** | **Bias** | **Bias** | **Bias** | **Bias** | **RRMISE** | **Bias** | **RRMISE** |
| N1 – Invasive | -0.023 | 0.581 | -0.008 | -0.012 | 0.007 | 0.007 | -0.011 | 0.580 | 0.007 | 0.579 |
| N2– ancestral Asia | 0.187 | 0.461 | 0.172 | 0.164 | 0.138 | 0.138 | 0.203 | 0.463 | 0.138 | 0.452 |
| N3 – ancestral Africa | 0.172 | 0.457 | 0.178 | 0.172 | 0.162 | 0.162 | 0.173 | 0.457 | 0.162 | 0.454 |
| t_1_inv - invasion | 0.024 | 0.520 | 0.085 | 0.081 | 0.042 | 0.042 | 0.046 | 0.533 | 0.042 | 0.541 |
| db –duration of bottleneck | 0.031 | 0.460 | 0.046 | 0.045 | -0.048 | -0.048 | 0.053 | 0.474 | -0.048 | 0.497 |
| N_1_b – effective number of founders | -0.009 | 0.474 | -0.017 | 0.021 | 0.057 | 0.057 | -0.018 | 0.481 | 0.057 | 0.501 |
| t_2_anc – ancestral divergence | 0.005 | 0.116 | 0.005 | 0.007 | 0.009 | 0.009 | 0.006 | 0.116 | 0.009 | 0.117 |
| ar - admixture | -0.003 | 0.169 | 0.003 | -0.000 | -0.008 | -0.008 | -0.005 | 0.161 | -0.008 | 0.212 |

| **Priors** | **Seville** | | **Greater London** | | **Rotterdam** | | **Hague** | | **Amsterdam** | | **Mauritius** | |
| --- | --- | --- | --- | --- | --- | --- | --- | --- | --- | --- | --- | --- |
|  | **Bias** | **Bias** | **Bias** | **Bias** | **Bias** | **Bias** | **Bias** | **RRMISE** | **Bias** | **RRMISE** | **Bias** | **RRMISE** |
| N1 – Invasive | -0.011 | -0.019 | 0.009 | -0.016 | -0.008 | -0.008 | -0.011 | 0.580 | 0.007 | 0.579 | -0.016 | 0.577 |
| N2– ancestral Asia | 0.203 | 0.195 | 0.219 | 0.144 | 0.169 | 0.169 | 0.203 | 0.463 | 0.138 | 0.452 | 0.144 | 0.445 |
| N3 – ancestral Africa | 0.173 | 0.192 | 0.199 | 0.162 | 0.173 | 0.173 | 0.173 | 0.457 | 0.162 | 0.454 | 0.162 | 0.455 |
| t_1_inv - invasion | 0.046 | 0.036 | 0.084 | 0.067 | 0.026 | 0.026 | 0.046 | 0.533 | 0.042 | 0.541 | 0.067 | 0.548 |
| db –duration of bottleneck | 0.053 | 0.033 | 0.081 | -0.023 | 0.001 | 0.001 | 0.053 | 0.474 | -0.048 | 0.497 | -0.023 | 0.501 |
| N_1_b – effective number of founders | -0.018 | -0.042 | -0.006 | 0.030 | 0.075 | 0.075 | -0.018 | 0.481 | 0.057 | 0.501 | 0.030 | 0.503 |
| t_2_anc – ancestral divergence | 0.006 | 0.009 | 0.013 | 0.004 | 0.004 | 0.004 | 0.006 | 0.116 | 0.009 | 0.117 | 0.004 | 0.115 |
| ar - admixture | -0.005 | 0.001 | 0.007 | -0.008 | -0.005 | -0.005 | -0.005 | 0.161 | -0.008 | 0.212 | -0.008 | 0.194 |

**Table S7.** Despite our efforts to collate as much information as possible from southern Europe, our dataset is biased towards more northerly parakeet populations. Concerning the analysis of proportion of haplotypes, the low sample size of southern populations makes our results dependent on the Seville population. We have repeated the analysis omitting each of the southern European populations, showing that only Tuscany can be removed from the dataset. Removing any other population results in P-values > 0.05.

| Population removed | R | P-value |
| --- | --- | --- |
| Seville | 0.41 | **0.17** |
| Marseille | 0.48 | **0.09** |
| Tuscany | 0.63 | 0.02 |
| Madrid | 0.45 | **0.12** |

**Table S8.** We carried out similar ‘sensitivity’ tests on our analyses of haplotypes prevalence or presence-absence in invaded Europe, showing that these analyses are robust concerning the inclusion of any southern European population.

| Haplotypes prevalence in invaded range | | | Haplotype presence/absence in the invaded range | | |
| --- | --- | --- | --- | --- | --- |
|  | R | P-value |  | R | P-value |
| Seville | 0.54 | 0.0055 | Seville | 3.74 | 0.0016 |
| Marseille | 0.54 | 0.0055 | Marseille | 3.74 | 0.0016 |
| Tuscany | 0.48 | 0.015 | Tuscany | 3.27 | 0.0041 |
| Madrid | 0.54 | 0.0055 | Madrid | 3.74 | 0.0016 |

Thus, our first finding that haplotypes with higher maximum native-range latitudes (i.e. a higher cold niche limit) are more prevalent in Europe/that haplotypes detected in Europe have a higher maximum native-range latitude than haplotypes not detected in Europe is not dependent on a specific southern European population. Our second finding that within European parakeet populations, the weighted average maximum native-range latitude of haplotypes present is positively and significantly correlated with the latitude of that population does however depend on specific southern European populations (i.e. only Tuscany can be excluded from the analysis).

**Table S9.** About the use of latitude as a proxy for temperature: we opted for latitude as a proxy to facilitate interpretation of Fig. 3.4.7, which illustrates that invasive populations originate predominantly from northern areas of the Asian native range. We derived the ‘minimum temperature of the coldest month’ from the WorldClim database (variable ‘bio6’) and find that in our dataset, minimum temperature and latitude are very strongly correlated (r = -0.97, P-value < 0.0001). Consequently, replacing latitude with minimum temperature does not affect our main conclusions. The table below compares the results obtained using latitude (as mentioned under the header *Influence of ‘native range climatic niche limits and the bird trade’*) with the results obtained using minimum temperature of the coldest month.

| Latitude | | Minimum temperature coldest month | |
| --- | --- | --- | --- |
| r: 0.54 | P-value: 0.0055 | r: -0.57 | P-value: 0.0028 |
| t: 3.74 | P-value: 0.0016 | t: -3.78 | P-value: 0.0013 |
| r: 0.54 | P-value: 0.048 | r: 0.54 | P-value: 0.047 |
